# 8oxoG:A Is Structurally Accommodated in the Nucleosome Core Particle, Yet Inaccessible to MUTYH-Initiated DNA Repair

**DOI:** 10.3390/biom16070999

**Published:** 2026-07-08

**Authors:** Abigayle F. Vito, Justin A. Ling, Julia C. Ferrara, Caleb S. Jacques, Natacha Gillet, Roy González-Alemán, Yuya Qiu, Mohammad Hashemian, Carlos H. Trasviña-Arenas, Sheila S. David, Sarah Delaney, Emmanuelle Bignon, Bret D. Freudenthal

**Affiliations:** 1 Department of Biochemistry and Molecular Biology, University of Kansas Medical Center, Kansas City, KS 66160, USA; 2 Department of Chemistry, Brown University, Providence, RI 02912, USA; 3 CNRS, ENS de Lyon, LCH, UMR 5182, 46 allée d’Italie, 69364 Lyon, France; 4 Université de Lorraine, CNRS, UMR 7019, LPCT, F-54000 Nancy, France; 5 Department of Chemistry, University of California, Davis, CA 95616, USA; 6 Department of Cancer Biology, University of Kansas Medical Center, Kansas City, KS 66160, USA; 7 University of Kansas Cancer Center, Kansas City, KS 66160, USA

**Keywords:** DNA repair, chromatin, mutagenesis, oxidative stress

## Abstract

Eukaryotic genomic DNA is packaged into chromatin as nucleosomes, where it remains susceptible to reactive oxygen species (ROS) that generate the mutagenic lesion 8-oxo-7,8-dihydroguanine (8oxoG). While 8-oxoguanine DNA glycosylase 1 (OGG1) can initiate repair of 8oxoG base paired with C within the nucleosome core particle (NCP) in a position- dependent manner, it is unknown whether MutY homolog (MUTYH), the DNA glycosylase that excises misincorporated A opposite 8oxoG, can initiate repair of 8oxoG:A base pairs within NCPs. To address this, we combined cryo-EM, molecular dynamics (MD) simulations, and biochemical assays. We determined that MUTYH activity on nucleosomal 8oxoG:A is strongly suppressed, with detectable excision limited to the entry/exit region. Cryo-EM structures at four superhelical locations reveal that 8oxoG adopts the *syn* conformation and Hoogsteen base pairs with A, as in non-nucleosomal DNA, indicating that lesion presentation is not altered by the histone octamer. MD simulations further reveal that 8oxoG:A base pair dynamics and local DNA backbone perturbations are similar in nucleosomal and non-nucleosomal DNA. Together, these data establish that the NCP sterically excludes MUTYH from 8oxoG:A base pairs, making them largely inaccessible to MUTYH processing. This work ultimately provides mechanistic insight for the elevated G to T transversion rate observed in histone-bound DNA following oxidative stress.

## 1. Introduction

In eukaryotes, genomic DNA is packaged into the nucleus in the form of chromatin, which consists of a fundamental repeating unit called the nucleosome. A nucleosome core particle (NCP) is comprised of ~147 base pairs of DNA wrapped around an octamer of histone proteins containing two copies of histones H2A, H2B, H3 and H4 [1,2]. Both nucleosomal and non-nucleosomal DNA are susceptible to several forms of DNA damage, one of the most common being oxidative damage [3,4,5,6,7,8]. Oxidative damage arises from excessive levels of reactive oxygen species (ROS) which can oxidize macromolecules in the cell [9,10]. The nucleobase G is highly susceptible to oxidation, forming the DNA lesion 8-oxo-7,8-dihydroguanine (8oxoG) [11]. If not repaired, 8oxoG can inappropriately base pair with adenine (A) during replication resulting in G to T (and complementary C to A) transversions, which are a common feature of cancer genomes with the single base substitution signature 18 (SBS 18) [12,13,14].

8oxoG lesions are primarily repaired through the base excision repair (BER) pathway [15]. BER is initiated by DNA glycosylases that recognize and excise the modified nucleobase. The primary DNA glycosylase responsible for identifying and removing 8oxoG is 8-oxo-guanine glycosylase 1 (OGG1) [16,17]. OGG1 specifically recognizes 8oxoG across from C and excises 8oxoG, leaving an abasic site that is further processed by downstream BER enzymes. If OGG1 does not identify and excise 8oxoG prior to DNA replication, replicative polymerases can insert dA complementary to 8oxoG, forming a mutagenic base pair [12]. A second DNA glycosylase, the human homolog of bacterial MutY (MUTYH), identifies 8oxoG:A base pairs and excises A opposite 8oxoG, resulting in an abasic site that is further processed by downstream BER enzymes. MUTYH achieves substrate specificity and performs its excision activity on non-nucleosomal (i.e., duplex DNA) by making extensive contacts with both DNA strands, including extrusion of A into its catalytic pocket and rotation of the opposing 8oxoG to the *anti* conformation through interactions with both its C- and N-terminal domains [18,19,20]. This engagement elicits a conformational change into a catalytically competent state in a manner similar to that observed with bacterial MutY with a variety of substrates [21,22,23,24,25]. Importantly, MUTYH-initiated BER allows the potential for restoration of the 8oxoG:C base pair which can then be recognized by OGG1, and repair can be initiated to avoid imprinting a mutation into the genome [17,26]. The importance of the repair of 8oxoG:A mismatches by MUTYH is underscored by the association of inherited variants of MUTYH and colorectal cancer, referred to clinically as MUTYH-associated polyposis (MAP) [17,24].

Chromatin structure has been shown to be a key determinant of oxidation-induced mutations in human genomes in which G to T mutations tend to persist in regions of tightly packed chromatin following oxidative stress [3,6,27]. This mutational signature is seemingly due to inefficient DNA repair as oxidative lesions were formed in a relatively uniform manner across tightly packed chromatin and linker DNA regions. Consistent with these observations, the compact nature of chromatin structure has been implicated in limiting the ability of DNA repair enzymes to access their DNA substrates [6,28,29,30]. DNA damage within the NCP is differentially accessible to repair enzymes depending on its position and prior studies suggest that accessibility of the DNA damage site dictates repair efficiency [31,32,33,34,35,36,37]. Indeed, recent studies have shown that OGG1 can access 8oxoG within an NCP at some solvent-exposed rotational orientations [6,31,32,38,39,40,41]. In contrast, whether MUTYH can access and repair 8oxoG:A lesions embedded within the NCP, and how 8oxoG impacts chromatin structure and dynamics, has not been determined. This gap represents a critical missing piece in our understanding of how oxidative lesions are accommodated and processed in chromatin.

To address this, we combined biochemical assays, cryo-EM, and classical MD simulations to determine MUTYH activity on nucleosomal 8oxoG:A lesions and to characterize the structure and dynamics of 8oxoG:A within the NCP. We find that MUTYH activity is strongly suppressed across the NCP, with detectable A excision restricted to the entry/exit region. Cryo-EM structures of NCPs containing 8oxoG:A at four distinct superhelical locations reveal that 8oxoG adopts a similar conformation to what is observed in non-nucleosomal DNA, indicating that the NCP does not alter the intrinsic base pairing properties of 8oxoG:A. MD simulations further reveal a dynamic behavior of the 8oxoG:A base pair, including a characteristic two-state conformational exchange and localized DNA backbone perturbations that are unchanged between nucleosomal and non-nucleosomal DNA. Together, these findings establish that the NCP sterically blocks MUTYH-initiated repair of the 8oxoG:A base pair, despite lesion presentation of 8oxoG being similar between nucleosomal and non-nucleosomal DNA.

## 2. Methods

### 2.1. Oligonucleotide Synthesis, Purification, and Preparation

For cryo-EM, all non-damaged oligonucleotides (oligos) were obtained from Integrated DNA Technologies (Coralville, IA, USA) and all oligos containing 8oxoG were obtained from TriLink BioTechnologies (San Diego, CA, USA). Each oligo was resuspended at 1 mM in a buffer containing 10 mM Tris-HCl (pH 7.5) and 1 mM EDTA. Each 8oxoG oligo was mixed with its non-damaged complement (see Supplementary Table S3) at equimolar ratios to a final concentration of 500 µM. The oligos were then annealed using a thermal cycler by heating to 95 °C then cooled to 4 °C at a rate of 0.5 °C min^−1^. The annealed oligos were immediately used for NCP reconstitution.

For biochemical studies, all DNA was synthesized using phosphoramidite chemistry on a Mermade4 DNA synthesizer from BioAutomation (Petaluma, CA, USA). Phosphoramidites were purchased from Glen Research (Sterling, VA, USA). 147-mer DNA was used for duplex controls and NCP samples (Figure S1). The complementary single strands are denoted as 601I (“I strand”) and 601J (“J strand”) based on the nomenclature used in an X-ray crystal structure of an NCP containing this Widom 601 DNA [42]. DNA nucleobases are labeled 1 to 147 in the 5′ to 3′ direction for the J strand. 8oxoG was globally incorporated into the I strand using previously reported methods to spike the T phosphoramidite with a small amount of 8oxoG phosphoramidite [43]. The ratio of T/8oxoG was determined by the Poisson distribution, modeled by the following equation:

$${P\left(k\right)=e}^{-\lambda }\frac{\lambda^{k}}{k!}$$

where P(*k*) is the probability of *k* lesions incorporated per strand, *k* is the number of lesions per strand, and *λ* is the average number of lesions per strand. By setting *λ* equal to 0.355, we ensure that 95% of the DNA contains, at most, one 8oxoG per strand, 25% contains one lesion per strand (P(1)), and 70% contains no lesions (P(0)). Both the I and J strands were cleaved from the solid support and protecting groups were removed by incubation with 30% (*v*/*v*) NH_4_OH at 55 °C for 19 h. The I strand (containing 8oxoG) cleavage and deprotection step was supplemented with 0.25 M β-mercaptoethanol (BME) to prevent oxidation of the lesion. The oligonucleotides were purified by 8% denaturing polyacrylamide gel electrophoresis (PAGE). The J strand (containing the A in the 8oxoG:A base pairs) was 5′-^32^P-radiolabeled and annealed to the 8oxoG-containing I strand in a buffer containing 10 mM Tris (pH 8) and 50 mM NaCl. A single-stranded 27-mer oligonucleotide was used as an internal standard and loading control (5′-GAT GTA TAT ATC TGA CAC GTG CTG GGA-3′). This internal standard was synthesized and purified as described above.

### 2.2. Purification of Recombinant Human Histones for Cryo-EM

Expression and purification of *H. sapien* histones were performed using well-established methods [44,45]. In brief, the genes encoding histones H2A, H2B, H3.2 (C110A), and H4 were previously cloned into a pet3a expression vector. The expression vectors were then transformed into T7 Express lysY competent cells (histone H2A, H3.2, H4; New England Biolabs, Ipswich, MA, USA) or BL21-CodonPlus competent cells (histone H2B; Agilent). The transformed cells were grown in M9 minimal media supplemented with a 1.0% vitamin solution at 37 °C until an OD_600_ of 0.4 was reached. Protein expression was induced with 0.4 mM IPTG (histone H2A, H2B, and H3) or 0.2 mM IPTG (histone H4) for 4 h (histone H2A and H2B) or 3 h (histone H3 and H4) at 37 °C. The cells were then harvested by centrifugation and resuspended in a buffer containing 50 mM Tris (pH 7.5), 100 mM NaCl, 5 mM BME, and 1 mM EDTA. The resuspended cells were lysed using sonication and inclusion bodies containing each histone were collected via centrifugation. The histones were extracted from the inclusion bodies under denaturing conditions in a buffer containing 20 mM Tris (pH 7.5), 6 M Guanidinium hydrochloride, and 10 mM dithiothreitol (DTT). The extracted histones were dialyzed into 8 M Urea and purified via subtractive anion-exchange and cation-exchange chromatography via gravity flow. The purified histones were dialyzed into H_2_O, lyophilized, and stored long term at −20 °C.

### 2.3. Preparation of H2A/H2B Dimers and H3/H4 Tetramers for Cryo-EM

H2A/H2B dimer and H3/H4 tetramer were generated and refolded using a previously established method [44,45]. In brief, each purified histone was resuspended in a buffer containing 20 mM Tris (pH 7.5), 6 M Guanidinium hydrochloride, and 10 mM DTT. To generate H2A/H2B dimer, equimolar amounts of histone H2A and H2B were combined, dialyzed three times against a buffer containing 20 mM Tris-HCl (pH 7.5), 2 M NaCl, 1 mM EDTA, and 1 mM DTT, and subsequently purified by gel filtration using a Sephacryl S-200 HR (Cytiva, Marlborough, MA, USA). To generate H3/H4 tetramer, equimolar amounts of histone H3 and H4 were combined, dialyzed three times against a buffer containing 20 mM Tris-HCl (pH 7.5), 2 M NaCl, 1 mM EDTA, and 1 mM DTT, and subsequently purified by gel filtration using a Sephacryl S-200 HR (Cytiva). The purified H2A/H2B dimer and H3/H4 tetramer were stored long-term at −20 °C in 50% glycerol.

### 2.4. NCP Reconstitution

For cryo-EM, NCPs were reconstituted using a previously established method [44,45]. In brief, the annealed 8oxoG DNA (see Supplementary Table S3), H2A/H2B dimer, and H3/H4 tetramer were combined in a 1:2:1 molar ratio in a buffer containing 20 mM Tris-HCl (pH 7.5), 2 M NaCl, 1 mM EDTA, and 1 mM DTT. The mixture was dialyzed stepwise against a no salt buffer stepwise to a final salt concentration of less than 150 mM NaCl over a period of 24 h. The reconstituted NCPs were subject to heat-shock at 55 °C for 30 min and subsequently purified via 10–40% sucrose gradient ultracentrifugation. The purified NCPs were stored short term (<1 month) at 4 °C.

For the biochemical assays, NCPs were reconstituted with canonical full-length human histone octamers purchased from The Histone Source at Colorado State University (Fort Collins, CO, USA) as previously described [46]. In brief, 1 µM 147-mer duplex and 1 µM histone octamer were combined in a 1:1 DNA:octamer molar ratio in a Slide-a-Lyzer dialysis device (0.1 mL capacity, 3.5 kDa MWCO; Thermo Fisher Scientific (Waltham, MA, USA)). Samples were incubated at 4 °C in a buffer containing 10 mM Tris-HCl (pH 7.5), 2 M NaCl, 1 mM EDTA, 1 mM DTT, and 0.5 mg/mL BSA. NaCl concentration was reduced stepwise at 1 h intervals (1.2, 1.0, 0.6 M) and 3 h interval (0 M) via dialysis. The EDTA concentration was reduced to 0 mM during the final hour of dialysis. NCPs were filtered by centrifugation using a Spin-X Centrifuge Tube filter (0.22 µm, Corning Incorporated (Corning, NY, USA)) to remove any precipitates. Samples were evaluated by 7% native PAGE (19:1 acrylamide:bisacrylamide, 4 °C, 2.5 h, 155 V, 0.25X Tris-borate-EDTA) to ensure that no excess duplex DNA was present (Figure S1).

### 2.5. Human MUTYH Expression and Purification

The overexpression and purification of MUTYH were performed as previously described [47]. Briefly, *E. coli* BL21 (DE3) co-transformed with the pRKISC and pKJE7 plasmids was used as the expression host. The pRKISC vector encodes the [4Fe-4S] cluster assembly machinery [48], while pKJE7 expresses the *dnaK*, *dnaJ*, and *grgE* chaperones [49]. Chemical competence was induced using RbCl to facilitate subsequent transformation with the pET28-MBP-MUTYH construct. Cells were recovered in SOC media for 1 h and plated on LB agar containing kanamycin (50 µg/mL), tetracycline (15 µg/mL), and chloramphenicol (34 µg/mL). Colonies were harvested to inoculate 2L of Terrific Broth supplemented with the same antibiotics in a 4 L flat-bottom flask. Cultures were grown at 37 °C with shaking at 180 rpm until reaching OD_600_ > 1.5 (~6 h), followed by cooling at 4 °C without shaking for 1 h. Protein expression was then induced with 1 mM IPTG, along with the addition of 0.1 g of ferrous sulfate and 0.1 g of ferric citrate. Expression proceeded at 15 °C for 16–24 h with shaking at 180 rpm. Two hours prior to harvesting, the cultures were supplemented with 0.120 g zinc sulfate heptahydrate. Cells were harvested by centrifugation (6000× *g*, 10 min, 4 °C) and stored at –80 °C.

For purification, frozen pellets were thawed and resuspended in lysis buffer (30 mM Tris-HCl [pH 7.5], 1 M NaCl, 20 mM 2-mercaptoethanol, and 10% glycerol) supplemented with 1 mM phenylmethylsulfonyl fluoride (PMSF). Cells were lysed by sonication on ice using 20 s on/40 s off cycles with 50% duty cycle and 0.5 amplitude (Branson Sonifier 250, Branson Ultrasonics Corporation, Danbury, CT, USA), followed by centrifugation at 15,000× *g* for 50 min at 4 °C. The clarified supernatant was incubated with 1.5 mL Ni^2+^-NTA resin (Qiagen, Hilden, Germany) for 1 h at 4 °C with rotation, then poured over a PD-10 column (Cytiva) for gravity flow.

The resin was washed with 25 mL lysis buffer and eluted with 10 mL elution buffer (30 mM Tris [pH 7.5], 200 mM NaCl, 30 mM 2-mercaptoethanol, 10% glycerol, and 500 mM imidazole). Prompt removal of imidazole and further purification was achieved via heparin affinity chromatography. The eluted sample was loaded onto a 1 mL Heparin column (Cytiva) pre-equilibrated with 10% buffer B (30 mM Tris [pH 7.5], 1 M NaCl, 10% glycerol, 1 mM DTT). Bound protein was eluted using a 10–100% linear gradient of buffer B over 45 min at 1.5 mL/min on an ÄKTA Pure 25 L (Cytiva). Fractions of MBP-MUTYH were quantified by UV-Vis to determine yield (MW = 100,888 g/mol, extinction coefficient = 152,470 M^−1^/cm^−1^) and collected for overnight proteolysis using TEV protease for the isolation of MUTYH from the MBP fusion protein. Finally, MUTYH was purified by a second heparin-affinity chromatography using an identical gradient and buffer system as the first heparin-affinity purification. Fractions containing pure MUTYH were assessed by SDS-PAGE and concentrated using Amicon centrifugal filters (10,000 MWCO). Protein concentration was determined by UV absorbance at 280 nm using an extinction coefficient of 84,630 M^−1^/cm^−1^. Aliquots were stored at –80 °C for future use.

### 2.6. MUTYH Excision Activity Assays

**Figure 1:**
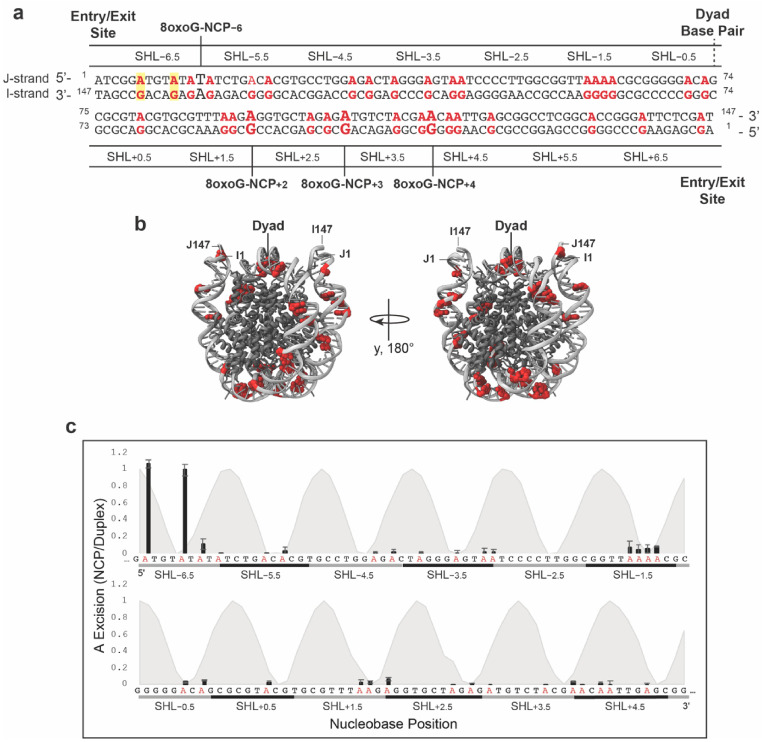
Excision of A from 8oxoG:A base pairs by MUTYH is limited to SHL−6.5 in nucleosomal DNA. (**a**) Diagram of the Widom 601 strong positioning sequence highlighting the globally incorporated 8oxoG:A base pairs. G shown in red represent 8oxoG. Every possible 8oxoG:A location is shown in red and the locations of observed excision of A by MUTYH are highlighted in yellow. Each of the 8oxoG containing strands contain at most one 8oxoG per NCP and will have a classical A:T base pair at all other sites. The J strand was 5′-^32^P-radiolabeled. (**b**) Two orientations of the nucleosome core particle (adapted from PDB: 7U51) with the 34 unique A positions highlighted in red. (**c**) Excision of A from 8oxoG:A in NCPs (A in the J strand). At each A site (shown in red) the product yield is expressed as the ratio of excision from the NCP relative to duplex after 120 min incubation with MUTYH (NCP/Duplex). The sequence is listed from 5′ to 3′ and the SHLs of the NCP are indicated. MUTYH activity in SHL+5.5 and SHL+6.5 was unable to be quantified due to insufficient resolution from the substrate. The gray shaded area shows the solution accessibility of each nucleobase, assigned from PDB 7U51. Error bars represent standard error (n = 3).

To assess MUTYH activity, 50 nM of either duplex or NCPs was incubated at 37 °C with 500 nM active MUTYH in 20 mM Tris (pH = 7.6), 50 mM NaCl, 150 mM KCl, 1 mM DTT, 0.2 mg/mL BSA in a total reaction volume of 82 µL. At various time intervals, a 20 µL aliquot was removed and quenched with 20 µL of 1 M NaOH for 2 min at 90 °C. The time intervals used were 1, 60, and 120 min for duplex and 60 and 120 min for NCPs. The 1 min time interval was excluded from the NCP sample to conserve material because our scouting experiments showed minimal MUTYH activity even at the later timepoints. A fresh aliquot of MUTYH (10 pmol) was added to the 120B sample after 60 min of incubation at 37 °C and incubated for an additional 60 min to account for the possibility that the lifetime of MUTYH could limit its activity. A negative-control sample (−E) was incubated in the absence of MUTYH at 37 °C and underwent the same quench and workup as other samples to reveal MUTYH-independent strand cleavage. DNA fragments were extracted from proteins with 25:24:1 phenol:chloroform:isoamyl alcohol and desalted by ethanol precipitation. Samples were resuspended in 50% aqueous formamide and loaded onto a 10% denaturing PAGE. Half of each sample was subject to electrophoresis for 3 h to resolve super-helical location (SHL) −1.5 to SHL+4.5, and the other half was subject to electrophoresis for 1.5 h to resolve SHL−6.5 to SHL−2.5. These positions span regions with differing strengths of DNA-histone interactions between the dyad and the entry/exit sites, enabling assessment of whether translational position influences MUTYH activity in the NCP (see Figure 1a,b). Importantly, these electrophoresis conditions were insufficient to resolve SHL+5.5 and SHL+6.5 from the substrate. Gels were imaged by phosphoimagery and quantified using GelAnalyzer 23.1.1 (available at www.gelanalyzer.com (accessed on 1 October 2024)) by Istvan Lazar Jr., PhD and Istvan Lazar Sr., PhD, CSc.

The band densities obtained by GelAnalyzer were normalized to an internal standard to account for uneven loading. Background damage was quantified via the negative control (−E) lane and subtracted from the MUTYH-treated samples. The amount of product accumulation at each 8oxoG:A site in the duplex at 120 min is defined as the theoretical maximum. To determine the relative A excision at the maximum time point (120 min) in the NCP, the normalized and background corrected pixel density at each 8oxoG:A position was divided by the pixel density, also normalized and background corrected, of the corresponding 8oxoG:A position at the longest time point (120 min) for the duplex control. The standard error was calculated using:

$$SE=\;\frac{\sigma }{\sqrt{n}}$$

where *σ* is the standard deviation and *n* is the number of experimental replicates (n = 3). To generate the solution accessibility profile (gray shaded region), PDB 7U51 was loaded into Pymol. An axis running through the center of mass of the histone octamer core in the x direction was created. For each nucleobase in the J strand, the distance was measured from the 5′-carbon atom to the central x axis. The accessibility of each nucleobase was established by first identifying the maximum and minimum 5′-carbon-to-axis distance within each helical turn. The accessibility value (A) was then calculated using:

$$A=\frac{\left(d-d_{min}\right)}{d_{max}}$$

where d is the 5′-carbon-to-axis distance value of the nucleobase, *d*_min_ is the minimum distance value within a helical turn and *d*_max_ is the maximum distance value within a helical turn. The values of A range from 1.0 (maximum accessibility) to 0.0 (minimum accessibility). Each normalized distance value was plotted as a function of nucleobase location.

### 2.7. Cryo-EM Sample and Grid Preparation

Nucleosomes containing 8oxoG:A at four distinct SHLs, SHL−6, SHL+4, SHL+3, and SHL+2, were generated for cryo-EM and are called 8oxoG:A-NCP−6, 8oxoG:A-NCP+4, 8oxoG:A-NCP+3 and 8oxoG:A-NCP+2, respectively. To generate the 8oxoG:A samples for cryo-EM, 12 µM of NCP containing 8oxoG damage across from A was mixed with 24 µM of MUTYH in a buffer containing 25 mM HEPES (pH 7.5), 50 mM NaCl, 0.5 mM TCEP, and 2.5 mM EDTA and incubated on ice for 10 min. Importantly, this sample was generated in attempt to capture a structure of MUTYH bound to the 8oxoG:A lesion and MUTYH is not required to obtain the 8oxoG:A-NCP structures presented here. The NCPs were crosslinked in a final concentration of 0.15% glutaraldehyde and incubated on ice for 20 min. Directly after incubation, the samples were loaded onto a Superdex S200 Increase 10/300 GL (Cytiva) that had been pre-equilibrated in a buffer containing 25 mM HEPES (pH 7.5), 50 mM NaCl, 0.5 mM TCEP, and 2.5 mM EDTA. The fractions containing NCP were identified via native polyacrylamide gel electrophoresis (5%, 59:1 acrylamide:bis-acrylamide). The samples were concentrated to 1.5 µM (8oxoG:A-NCP−6 and 8oxoG:A-NCP+4) or 2 µM (8oxoG:A-NCP+3 and 8oxoG:A-NCP+2). Gels for each 8oxoG:A-NCP sample used for cryo-EM grid preparation can be found in Supplemental Figures S5 and S6.

Nucleosomes containing 8oxoG:C at two distinct SHLs, SHL+3, and SHL+2, were generated for cryo-EM and are called 8oxoG:C-NCP+3 and 8oxoG:C-NCP+2, respectively. To generate the 8oxoG:C samples for cryo-EM, 5 µM of reconstituted NCPs containing 8oxoG damage across from C was mixed with 7.5 µM–10 µM of OGG1 K249Q in a buffer containing 25 mM HEPES (pH 7.1), 25 mM NaCl, 1 mM TCEP and 1 mM EDTA and incubated on ice for 10 min. Importantly, this sample was generated in attempt to capture a structure of OGG1 bound to the 8oxoG:C lesion and OGG1 is not required to obtain the 8oxoG:C-NCP structures presented here. The NCPs were crosslinked in a final concentration of 0.1% glutaraldehyde and incubated on ice for 20 min. Directly after incubation, the samples were loaded onto a Superdex 200 Increase 10/300 GL (Cytiva) that had been pre-equilibrated in a buffer containing 50 mM HEPES (pH 7.1), 100 mM NaCl, 1 mM TCEP, and 1 mM EDTA. The fractions containing NCP were identified via native polyacrylamide gel electrophoresis (5%, 59:1 acrylamide:bis-acrylamide). The samples were concentrated to 1.5 µM for short-term storage. Gels for each 8oxoG:C-NCP sample used for cryo-EM grid preparation can be found in Supplemental Figure S15. 3 µL of each concentrated sample for both 8oxoG:A-NCP and 8oxoG:C-NCP samples was applied to a Quantifoil R2/2 300 mesh copper cryo-EM grid and plunge frozen in liquid ethane using a Vitrobot Mark IV (Thermo Fisher). The cryo-EM grids were clipped and stored in liquid nitrogen prior to screening and data collection.

### 2.8. Cryo-EM Data Collection and Processing

The cryo-EM data collections for 8oxoG:C-NCP+3 and 8oxoG:C-NCP+2 were performed using a Titan Krios G3i with Gatan K3 camera and BioQuantum energy filter at the University of Chicago Advanced Electron Microscopy Core Facility (Chicago, IL, USA) with a raw pixel size of 0.534 Å/pixel. The cryo-EM data collections for 8oxoG:A-NCP−6, 8oxoG:A-NCP+4, 8oxoG:A-NCP+3, and 8oxoG:A-NCP+2 were performed using a Titan Krios with Falcon 4i camera and Selectris X energy filter at Pacific Northwest Cryo-EM Center (Portland, OR, USA) with a raw pixel size of 0.394 Å/pixel (8oxoG:A-NCP−6, 8oxoG:A-NCP+4, 8oxoG:A-NCP+3) or 0.4125 Å/pixel (8oxoG:A-NCP+2). The cryo-EM datasets were each processed using cryoSPARC v4 (Toronto, ON, Canada) [50,51] and the complete processing workflow for each dataset is shown in Supplemental Figures S5, S6 and S15. In brief, the micrographs were corrected for particle motion resulting from stage drift and beam irradiation using Patch Motion Correction and subsequently contrast transfer function (CTF) fit using Patch CTF. The micrographs were then manually curated to exclude micrographs based on factors including CTF fit resolution and ice thickness. A random subset of the curated micrographs was then used to generate initial 2D classes via blob picker, and these initial 2D classes were used for automated template picking of the complete dataset. The template picked particles were curated and extracted from the micrographs at a box size of 600 pixels and down sampled to 256 pixels. The extracted particles were subjected to multiple rounds of 2D classification to yield a final particle stack. The final particle stacks were used to generate an ab initio model followed by multiple rounds of heterogeneous refinement to separate out destroyed or unwrapped NCPs. For all datasets apart from 8oxoG:A-NCP+2, 3D classification was performed to improve the interpretability of the map using a focus mask for the entry/exit site DNA, which is often found unwrapped from the histone core. The final particles were re-extracted at a full box size of 600 pixels and subjected to local CTF refinement and final non-uniform refinement. The final maps were deposited into the Electron Microscopy Data Bank (EMDB) under accession numbers EMD-75155 for 8oxoG:A-NCP+2, EMD-75154 for 8oxoG:A-NCP+3, EMD-75153 for 8oxoG:A-NCP+4, and EMD-75152 for 8oxoG:A-NCP−6, EMD-75157 for 8oxoG:C-NCP+2, and EMD-75156 for 8oxoG:C-NCP+3.

### 2.9. Model Building and Refinement

Model building and refinement for each structure was performed using the University of California San Francisco (San Francisco, CA, USA) ChimeraX 1.7, PHENIX 1.22 (Berkeley, CA, USA), and COOT 0.9.9 (Cambridge, UK) [52,53,54,55,56]. A cryo-EM NCP structure containing an AP-site at SHL−6 (PDB: 7U51) [28] was used as a starting model for each structure and 8oxoG and A (if applicable) were subsequently built in at the locations of damage. The models for each structure were docked into its respective cryo-EM map using ChimeraX. The models were then iteratively refined in PHENIX using secondary structure restraints and manual adjustments were performed to the model using COOT. The final models were validated using MolProbity [57] and deposited into the Protein Data Bank (PDB) under accession numbers 10GM for 8oxoG:A-NCP+2, 10GL for 8oxoG:A-NCP+3, 10GK for 8oxoG:A-NCP+4, 10GJ for 8oxoG:A-NCP−6, 10GO for 8oxoG:C-NCP+2, and 10GN for 8oxoG:C-NCP+3. All depictions of the maps and models presented in the manuscript were generated using UCSF ChimeraX.

### 2.10. Molecular Dynamics Simulations

Cryo-EM structures were used as starting structures for molecular dynamics simulations of 8oxoG:C-NCP+2, 8oxoG:C-NCP+3, 8oxoG:A-NCP−6, 8oxoG:A-NCP+4, 8oxoG:A-NCP+3, and 8oxoG:A-NCP+2. In addition, 8oxoG:C-NCP+4, 8oxoG:C-NCP−6 and control non-damaged NCP (ND-NCP) structures were computationally created starting from the 8oxoG:C-NCP+2 structure. A 21 bp non-nucleosomal, double-stranded DNA sequence was built as the starting model for a control system with the sequence 5′-CGG CTG TAT AG*A TCT GAC AGC-3′ and its complementary sequence (*indicates site of 8oxoG in damaged system). Histone tails were added in an extended conformation after alignment of the NCP with the 1KX5 PDB structure [58]. Five starting conformations of the tails were thus designed per sequence. Each system was solvated in a truncated octahedron box of TIP3P water [59] with a buffer of 15 Å around the solute. Sodium cations and chloride anions were added to reach neutrality and a physiological saline concentration of 0.10 M.

The MD simulations were performed using Amber20 facilities [60]. Protein and DNA were described using ff14SB [61] and parmbsc1 force field [62] force fields respectively. The 8oxoG force field was taken from literature [63]. Protonation states have been determined according to PropKa calculations [64]. CUFIX correction was applied to improve ionic interactions [65]. The hydrogen mass repartitioning (HMR) method [66] was used to allow a timestep of 4 fs for all equilibration and production trajectories. All simulations were run in periodic boundary conditions with a cut-off of 10 Å for non-covalent interactions and the Particle-Mesh Ewald approach for electrostatic interactions. First, all systems were minimized with 5000 steps of steepest descent followed by 5000 steps using conjugate gradient. Then, they were heated from 0 K to 300 K during 30 ps, with a timestep of 1 fs, using a Langevin thermostat with a collision frequency of 1 ps^−1^ in the NVT ensemble. An equilibration run at 300 K was performed during 100 ns, with a pressure of 1 atm using the Berendsen barostat. Finally, a production of 1 μs was performed for each system, totaling 5 replicas per sequence. The same protocol was applied to model the 8oxoG:C and 8oxo:A in duplex DNA. Starting structure consisting of a 21 base pairs oligonucleotide centered on the lesion were extracted from the cryo-EM structures featuring the lesion at SHL−6, and 3 replicas of 1 µs were collected for each system (8oxoG:C, 8oxoG:A and undamaged control). All MD data is available online on a Zenodo repository (https://doi.org/10.5281/zenodo.14755037).

Base pair parameters were computed using the courbes program [67]. This Python3 wrapper uses Curves+ [68] as a backend and automates the extraction of critical descriptors from multiple replicas of molecular dynamics simulations, generating basic statistical reports and facilitating efficient data analysis. Courbes is freely available at https://github.com/rglez/courbes (accessed on 1 April 2025). The radial distributions of water molecules and sodium ions were computed with the cpptraj program of Amber20.

## 3. Results

### 3.1. MUTYH Is Not Appreciably Active in the NCP

To test MUTYH excision activity in an NCP, we performed biochemical experiments on a population of NCPs in which each NCP contains at most one 8oxoG:A base pair. Generation of this population is enabled by a DNA chemical synthesis strategy we previously reported [43]. We generated a modified Widom 601 sequence so that the “I” strand contains 8oxoG and the complementary 5′-^32^P-radiolabeled “J” strand contains A (Figure 1a). During synthesis of the I strand, a small amount of the 8oxoG phosphoramidite is included with the T version, yielding DNA that allows for preparation of NCPs with 8oxoG:A at 34 unique locations spanning all super-helical locations (SHLs) (Figure 1a,b). This strategy provides the functional equivalent of individually synthesizing 34 8oxoG-containing oligonucleotides, each differing by only the lesion position. The A nucleobase is always located on the same strand of DNA but exhibits varying levels of solvent exposure and accessibility depending on position within the NCP (Figure 1, Table S1). The population of NCPs was incubated with excess MUTYH under single-turnover conditions. Abasic sites generated by the excision of A by MUTYH were converted to strand breaks by NaOH treatment and visualized by denaturing PAGE as previously described (Figure S2) [32,43]. We used the same population of DNA containing 8oxoG:A, but not bound to histones, as a positive control for MUTYH activity on non-nucleosomal DNA.

MUTYH efficiently excised A from all 31 locations in the non-nucleosomal (i.e., duplex) DNA substrates that could be sufficiently resolved by denaturing PAGE. (Figure S2, red dashes). MUTYH activity at the three A sites within SHL+5.5 and SHL+6.5 generates strand breaks that result in fragments of sufficiently large size, preventing resolution from the substrate (Figure S2). Moreover, a similar amount of strand breakage at each A site, indicated by a similar band density, suggests comparable activity at each position in non-nucleosomal DNA under single turnover conditions (Figure S2a,b). In contrast, the band density at all but two A locations in nucleosomal DNA is indistinguishable from the no enzyme (−E) negative controls (Figure 1c and Figure S2). This result indicates that MUTYH does not excise A from the vast majority of nucleosomal 8oxoG:A lesions despite the presence of solvent-exposed A (Table S1). Detectable MUTYH activity was observed at two positions in the NCP, both located at SHL−6.5 (Figure 1c and Figure S2b; yellow highlights). At this position, the DNA is in the entry/exit region of the NCP and is susceptible to transient and spontaneous unwrapping [69]. We envision that the increased accessibility of DNA in the entry/exit region likely enables MUTYH to form the extensive contacts with both strands of DNA required for catalysis, similar to those observed in non-nucleosomal DNA.

### 3.2. Intrinsic Structure and Dynamics of 8oxoG:A Are Preserved in the NCP

**Figure 2:**
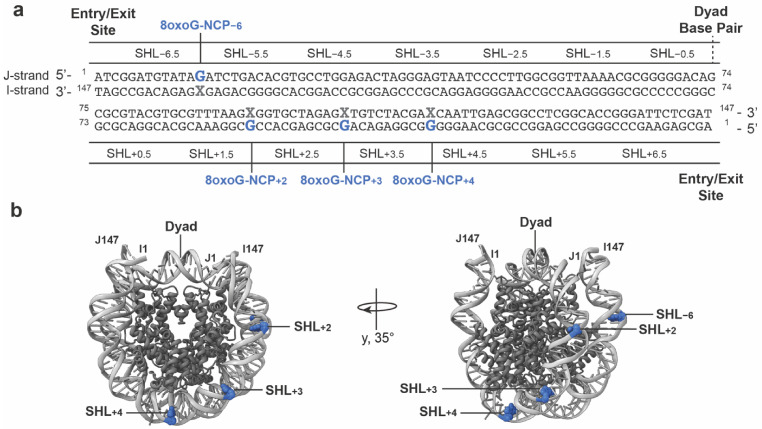
Translational positions of nucleosomal 8oxoG for structural analysis. (**a**) Diagram of the Widom 601 strong positioning sequence showing the 8oxoG sites used to reconstitute damaged NCPs. 8oxoG sites are shown in blue and their complementary bases (C or A) are shown with a gray X. (**b**) Two orientations of the NCP (PDB: 7U51) with 8oxoG positions at SHL+2, SHL+3, SHL+4, and SHL−6 labeled.

The reduced activity of MUTYH on NCP-embedded 8oxoG:A lesions motivated efforts to capture a stable MUTYH-NCP complex to provide insight into the structural basis for this reduced activity. Despite extensive attempts to determine a cryo-EM structure of MUTYH bound to 8oxoG:A-containing NCPs at multiple positions, no stable complexes were obtained. We therefore sought to determine whether lesion presentation within chromatin might underlie the observed lack of MUTYH activity by investigating the structure and dynamics of NCPs containing 8oxoG opposite A. To this end, we generated NCPs containing site-specific 8oxoG lesions within the 147 bp Widom 601 strong positioning sequence and determined cryo-EM structures of NCPs containing 8oxoG:A at four distinct SHLs (SHL+2, SHL+3, SHL+4, and SHL−6; Figure 2). These positions span regions with differing strengths of DNA-histone interactions between the dyad and the entry/exit sites, enabling assessment of whether translational position influences base pairing properties or overall NCP structure.

**Figure 3:**
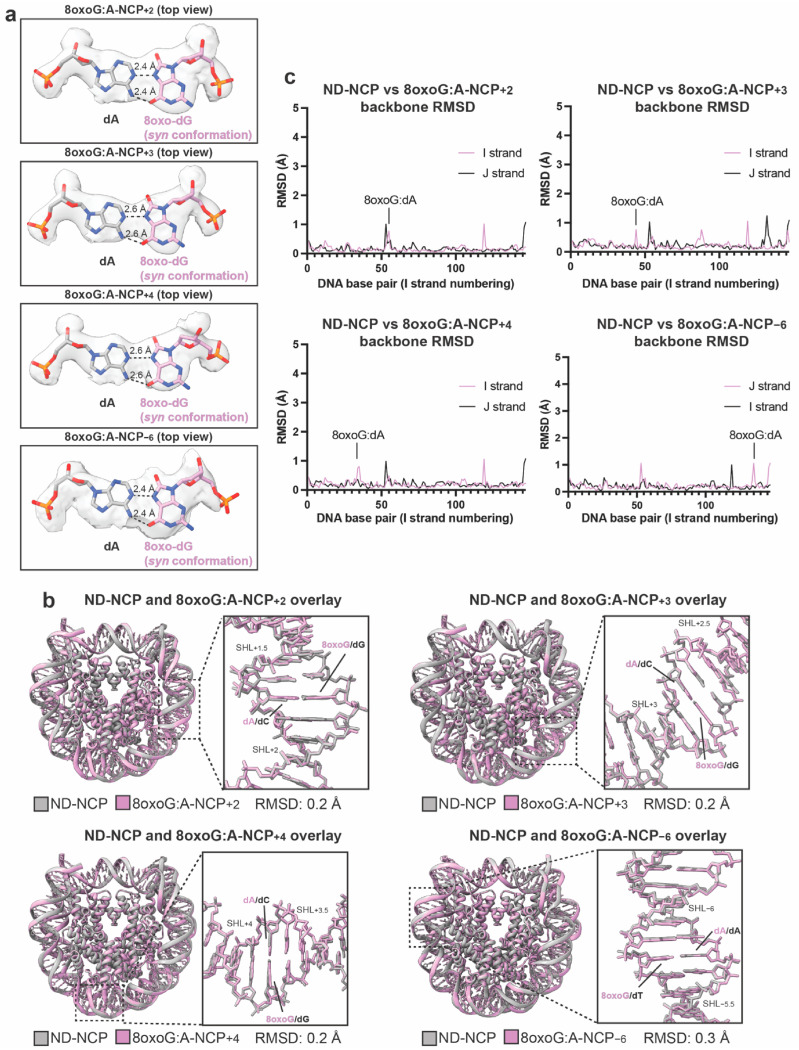
Accommodation of the 8oxoG:A base pair in the NCP is the same across multiple translational positions. (**a**) Top view of the 8oxoG:A base pair at SHL+2, SHL+3, SHL+4, and SHL−6. The density from the cryo-EM map is shown as a transparent gray surface. The dotted lines represent hydrogen bonds between bases and are labeled with their respective lengths. (**b**) Structural comparison of the ND-NCP (gray, PDB: 10XZ) and 8oxoG:A-NCP at SHL+2, SHL+3, SHL+4, and SHL−6 (pink). RMSD value represents the differences between the histone octamers. (**c**) Per-base-pair comparison of the DNA backbone between ND-NCP (PDB: 10XZ) and 8oxoG:A-NCP at SHL+2, SHL+3, SHL+4, and SHL−6. The strand containing 8oxoG is shown in pink and the strand containing A is shown in black. The x-axis is numbered from base pair 1-147 according to I-strand numbering and the location of the 8oxoG:A base pair is labeled.

To obtain insight into the structure of the 8oxoG:A base pair in the NCP, we generated recombinant NCPs containing an 8oxoG:A base pair at SHL+2 (8oxoG:A-NCP+2), SHL+3 (8oxoG:A-NCP+3), SHL+4 (8oxoG:A-NCP+4), and SHL−6 (8oxoG:A-NCP−6) (Figure S3). At SHL+2, SHL+3, and SHL+4, the 8oxoG:A base pair is positioned two, three, and four superhelical turns away from the NCP dyad, respectively. At SHL−6, the 8oxoG:A base pair is near the entry exit site on the opposite face of the NCP (Figure 2b). At each position, 8oxoG is solvent-exposed and the opposing A is facing towards the histones (i.e., histone-occluded). We performed single particle analysis and determined a 2.5 Å, 2.5 Å, 2.7 Å, and 2.8 Å cryo-EM reconstruction of 8oxoG:A-NCP+2, 8oxoG:A-NCP+3, 8oxoG:A-NCP+4, and 8oxoG:A-NCP−6, respectively (Figures S4–S6, Table S2). All four reconstructions were of sufficient quality to unambiguously assign the DNA register and define the position and geometry of the 8oxoG:A base pair (Figures S7–S10). In all four structures, 8oxoG adopts the *syn* conformation and forms a Hoogsteen base pair with A (Figure 3a). This conformation matches that observed in a high-resolution x-ray crystal structure of 8oxoG:A in non-nucleosomal DNA [70], indicating that NCP structure and the translational position of 8oxoG:A do not significantly alter the intrinsic base pairing conformation of 8oxoG:A. To assess potential effects of the presence of 8oxoG:A on global NCP structure, we performed structural comparison of each 8oxoG:A-NCP structure to a recently reported 2.6 Å non-damaged NCP (ND-NCP) containing the same Widom 601 sequence besides the 8oxoG lesion (PDB: 10XZ) [71]. These comparisons revealed minimal changes to the histone octamer for 8oxoG:A-NCP+2 (RMSD: 0.2 Å), 8oxoG:A-NCP+3 (RMSD: 0.2 Å), 8oxoG:A-NCP+4 (RMSD: 0.2 Å), and 8oxoG:A-NCP−6 (RMSD: 0.3 Å), compared to the ND-NCP (Figure 3b). Similarly, the presence of 8oxoG:A caused minimal changes to the nucleosomal DNA with per-base-pair RMSD values across the 147-bps remaining near or below 1 Å relative to the ND-NCP (Figure 3c). Together, these data indicate that the presence of an 8oxoG:A base pair at the positions tested does not impact global NCP structure. Notably, our biochemical data showed no detectable A excision by MUTYH at SHL+2, SHL+3, or SHL+4, corresponding to the positions captured structurally in the 8oxoG:A-NCP+2, 8oxoG:A-NCP+3, and 8oxoG:A-NCP+4 structures, respectively. Although our biochemical assay did not directly contain the SHL−6 position from our structure, MUTYH activity was not observed at adjacent locations within SHL−6 (see above). Interestingly, our 8oxoG:A-NCP structures show that the 8oxoG:A base pair does not cause significant distortion in the DNA that may facilitate recognition and excision of A by MUTYH.

**Figure 4:**
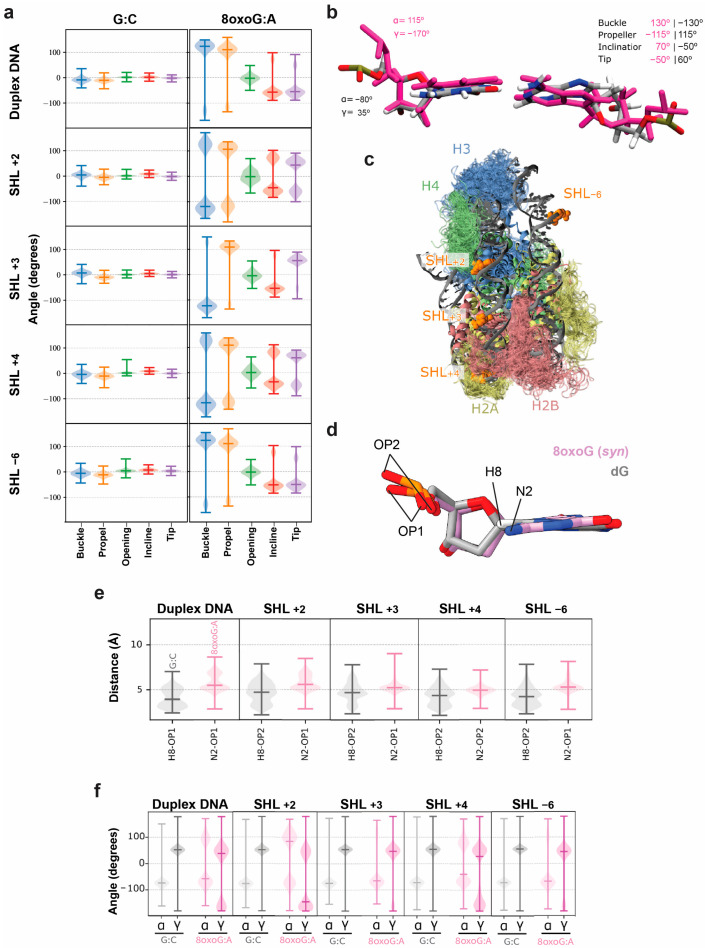
Dynamics of the 8oxoG:A base pair in nucleosomal and non-nucleosomal (duplex) DNA. (**a**) Distribution of the intra-base pair angles for the control and 8oxoG:A systems in duplex DNA or in the NCP at SHL+2, SHL+3, SHL+4, and SHL−6. (**b**) Superimposition of representative snapshots from the simulation with 8oxoG:A., illustrating the two conformations sampled for the 8oxoG:A mismatch. The conformations feature distinct backbone angles and intra-base pair parameter values, some of which are displayed. Structures have been aligned on the nucleotides sugar rings to show the backbone and nucleobase dynamics. (**c**) Side view of the projection of histone tail conformations sampled by MD simulations. The SHLs of the lesions are labeled in orange. (**d**) Superimposition of G (gray) and 8oxoG in the *syn* conformation (pink). Non-bridging oxygen atoms on the phosphate group (OP1 or OP2) and the closest atom on the nucleobase (H8 for G or N2 for 8oxoG) are labeled. (**e**) Distribution of the difference between either the H8 (undamaged, gray) or N2 (for 8oxoG:A, pink) atom and the closest non-bridging oxygen atom on the phosphate group (OP1 or OP2) calculated from MD simulations in duplex DNA or in the NCP at SHL+2, SHL+3, SHL+4, and SHL−6. (**f**) Distribution of the α and γ backbone angles of 8oxoG or dG in the control, for duplex DNA or in the NCP at SHL+2, SHL+3, SHL+4, and SHL−6.

The 8oxoG:A-NCP cryo-EM structures represent a stable, single conformation of the 8oxoG:A base pair and NCP structure. To further characterize the lesion dynamics, we performed five replicates of 1 µs classical molecular dynamics (MD) simulations on the 8oxoG:A-NCP+2, 8oxoG:A-NCP+3, 8oxoG:A-NCP+4, and 8oxoG:A-NCP−6 structures. Additionally, we performed five replicates of 1 µs classical MD simulations on the ND-NCP and three replicates of 1 µs MD simulations on a 21 bp non-nucleosomal (i.e., duplex) DNA oligonucleotide with and without an 8oxoG:A base pair. We found that in the ND-NCP and non-damaged duplex systems, the G:C base pair exhibits a monotonous dynamic behavior and maintains classical Watson–Crick base pairing, whereas in all 8oxoG:A systems, the 8oxoG:A Hoogsteen base pair exhibited a specific dynamic behavior within the DNA helix (Figure 4a). Analysis of the intra-base pair parameters of 8oxoG:A revealed a rapid exchange between two states, characterized by distinct values of the buckle, propeller, inclination, and tip angles: 130°/−115°/70°/−50° respectively for the first state, and −130°/115°/−50°/60° respectively for the second state (Figure 4b). The presence of two distinct states was consistent across non-nucleosomal and nucleosomal DNA containing 8oxoG:A, indicating that the NCP does not alter the intrinsic dynamic nature of the 8oxoG:A base pair. Subtle differences in the distribution of the two states were observed depending on the position in the NCP which could be attributed to the environment of the base pair, namely the local DNA sequence and/or the exposure to interactions with histone tails in the nucleosome (Figure 4c). Despite this dynamic behavior, hydrogen bonds between the nucleobases were stable along the simulations and perturbations were highly localized. Dynamic behavior on the adjacent base pairs was limited to a bimodal distribution of only the 5′ base pair tip angle (Figure S11). Additionally, in agreement with the cryo-EM structures, the MD trajectories showed that global NCP dynamics were not impacted by the presence of an 8oxoG:A base pair. However, local perturbations of the DNA backbone were observed at the 8oxoG:A base pair to accommodate the extra O8 atom present in 8oxoG. In each 8oxoG:A-NCP system, the distance between the N2 atom of 8oxoG and the closest non-bridging oxygen on the phosphate (OP1 or OP2) was greater than that for the H8 atom of G and the closest non-bridging oxygen in the ND-NCP system (Figure 4d,e), reflecting altered local DNA backbone geometry due to the presence of 8oxoG:A. Importantly, this local rearrangement of the backbone is observed in both non-nucleosomal and nucleosomal DNA, indicating that the NCP does not alter the local DNA backbone perturbation. In nucleosomal and non-nucleosomal DNA, the presence of the O8 atom of 8oxoG induces the formation of an organized first solvation shell around this atom, which is absent in the ND-NCP system. One or two water molecules can bridge O8 of 8oxoG and the DNA backbone (Figure S12). Similarly, sodium cations are present within 3 Å of the O8 of 8oxoG atom (Figure S13), and these interactions can impact the DNA backbone angle distribution. In the Hoogsteen configuration, the O8 atom of 8oxoG points towards the minor groove, which promotes interactions with bridging water molecules and sodium cations. For this reason, water molecules and sodium cations within 3 Å of O8 were found in higher quantities in the 8oxoG:A systems as compared to the non-damaged control systems (Figures S12 and S13). To determine whether these interactions altered the DNA backbone conformation of 8oxoG compared to the canonical G nucleobase, we examined the distribution of α and γ backbone angles across the non-damaged and 8oxoG:A systems. We found that the non-damaged systems exhibit a single distribution of α and γ backbone angles, whereas all 8oxoG:A systems show a bimodal distribution of α and γ backbone angles, indicating the presence of two distinct backbone conformations (Figure 4f and Supplementary Video). These local rearrangements of the DNA backbone were also observed in the non-nucleosomal duplex DNA containing 8oxoG:A, though to slightly different extents, likely due to the local environment of the lesion. Collectively, these findings indicate that 8oxoG:A induces localized DNA backbone perturbations that are largely unchanged between nucleosomal and non-nucleosomal DNA. Overall, our structural and MD simulation data indicate that 8oxoG:A maintains its intrinsic base pairing properties and dynamic behavior within the NCP and does not induce significant distortion of the NCP architecture, suggesting that these features do not account for the reduced MUTYH activity on NCP substrates.

### 3.3. Intrinsic Structure and Dynamics of 8oxoG:C Are Preserved in the NCP

To further probe how the NCP influences 8oxoG lesion presentation, we next examined the structure and dynamics of the 8oxoG lesion base paired to C. This analysis serves as an essential comparison to the 8oxoG:A structure and dynamics and provides a complete picture of lesion presentation of 8oxoG within the NCP.

**Figure 5:**
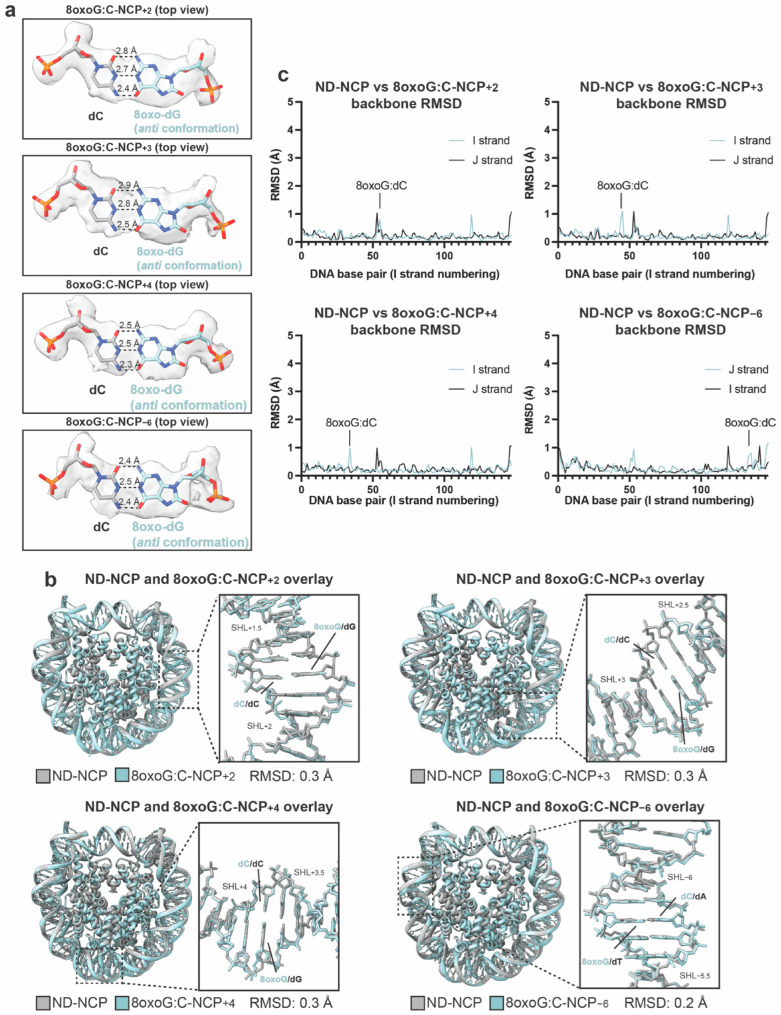
Accommodation of the 8oxoG:C base pair in the NCP is the same across multiple translational positions. (**a**) Top view of the 8oxoG:C base pair at SHL+2, SHL+3, SHL+4 (PDB: 8VWU), and SHL−6 (PDB: 8VWS). The density from the cryo-EM map is shown as a transparent gray surface. The dotted lines represent hydrogen bonds between bases and are labeled with their respective lengths. (**b**) Structural comparison of the ND-NCP (gray, PDB: 10XZ) and 8oxoG:C-NCP at SHL+2, SHL+3, SHL+4, and SHL−6 (blue). RMSD value represents the differences between the histone octamers. (**c**) Per-base-pair comparison of the DNA backbone between ND-NCP (PDB: 10XZ) and 8oxoG:C-NCP at SHL+2, SHL+3, SHL+4, and SHL−6. The strand containing 8oxoG is shown in blue and the strand containing C is shown in black. The x-axis is numbered from base pair 1-147 according to I-strand numbering and the location of the 8oxoG:C base pair is labeled.

To obtain insight into the structure of the 8oxoG:C base pair in the NCP, we generated recombinant NCPs containing an 8oxoG:C base pair at SHL+2 (8oxoG:C-NCP+2) and SHL+3 (8oxoG:C-NCP+3), (Figure S3). At both positions, 8oxoG is solvent-exposed and the opposing C is histone-occluded. We performed single particle analysis and obtained a 2.8 Å and 3.0 Å cryo-EM reconstruction of 8oxoG:C-NCP+2 and 8oxoG:C-NCP+3, respectively (Figures S14 and S15, Table S2). Importantly, both reconstructions were of sufficient quality to readily assign the register of the nucleosomal DNA and accurately define the position and geometry of the 8oxoG:C base pair (Figures S16 and S17). In both structures, 8oxoG adopts the *anti* conformation and forms classic Watson–Crick base pairing with C (Figure 5a). In addition, we previously reported two NCP structures containing 8oxoG:C at SHL+4 (8oxoG:C-NCP+4) and SHL−6 (8oxoG:C-NCP−6) at resolutions of 3.0 Å and 3.1 Å, respectively (Figure S14e,f) [6]. Both structures feature a solvent-exposed 8oxoG and histone-occluded opposing C. Importantly, in both previously reported structures, the 8oxoG lesion adopts the *anti* conformation and forms classic Watson–Crick base pairing with C (Figure 5a). This conformation matches that observed in a high-resolution x-ray crystal structure of an 8oxoG:C base pair in non-nucleosomal DNA [72] and in our 8oxoG:C-NCP+2 and 8oxoG:C-NCP+3 structures, indicating that NCP structure and the translational position of the lesion does not alter the base pairing properties of 8oxoG:C. To determine whether the 8oxoG:C base pair impacts overall NCP structure, we performed a structural comparison of each 8oxoG:C-NCP structure to the ND-NCP (PDB:10XZ). This analysis revealed minimal changes to the histone octamer for 8oxoG:C-NCP+2 (RMSD: 0.3 Å), 8oxoG:C-NCP+3 (RMSD: 0.3 Å), 8oxoG:C-NCP+4 (RMSD: 0.3 Å) and 8oxoG:C-NCP−6 (RMSD: 0.2 Å) compared to the ND-NCP (Figure 5b). Similarly, the presence of 8oxoG:C caused minimal changes to the nucleosomal DNA, with per-base-pair RMSD values across the 147-bps remaining near or below 1 Å relative to the ND-NCP (Figure 5c). Cumulatively, these data indicate that the presence of an 8oxoG:C base pair at the positions tested does not alter the global NCP structure.

**Figure 6:**
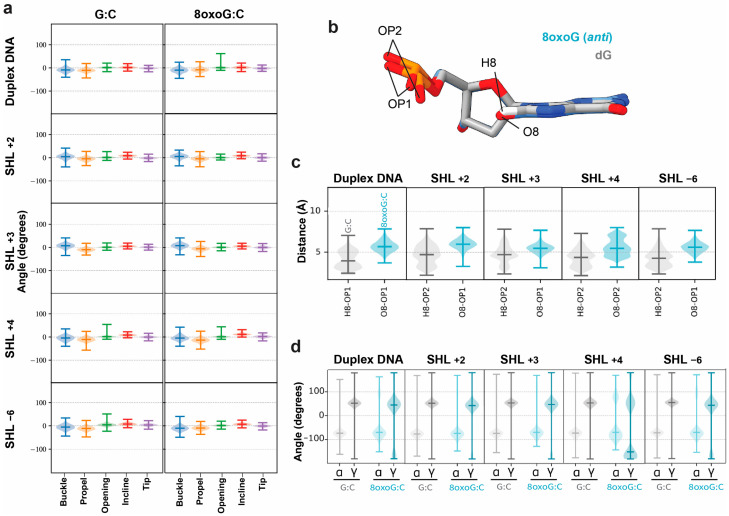
Dynamics of the 8oxoG:C base pair in nucleosomal and non-nucleosomal (duplex) DNA. (**a**) Distribution of the intra-base pair angles for the control and 8oxoG:C systems in duplex DNA or in the NCP at SHL+2, SHL+3, SHL+4, and SHL−6. (**b**) Superimposition of G (gray) and 8oxoG in the *anti* conformation (blue). Non-bridging oxygen atoms on the phosphate group (OP1 or OP2) and the closest atom on the nucleobase (H8 for G or O8 for 8oxoG) are labeled. (**c**) Distribution of the difference between either the H8 (undamaged, gray) or O8 (for 8oxoG:C, blue) atom and the closest non-bridging oxygen atom on the phosphate group (OP1 or OP2) calculated from MD simulations in duplex DNA or in the NCP at SHL+2, SHL+3, SHL+4, and SHL−6. (**d**) Distribution of the α and γ backbone angles of 8oxoG or dG in the control, for duplex DNA or in the NCP at SHL+2, SHL+3, SHL+4, and SHL−6.

To characterize the dynamics of the 8oxoG:C lesion within the NCP, we performed five replicates of 1 µs classical MD simulations on the 8oxoG:C-NCP+2, 8oxoG:C-NCP+3, 8oxoG:C-NCP+4, and 8oxoG:C-NCP−6 structures and three replicates of 1 µs classical MD simulations on a 21 bp duplex DNA oligonucleotide with and without an 8oxoG:C base pair. Analysis of the intra-base pair structural parameters reveals that in all 8oxoG:C-NCP systems, the 8oxoG:C base pair exhibits a monotonous dynamical behavior and maintains classical Watson–Crick base pairing, similar to the non-damaged systems (Figure 6a). This trend is consistent across non-nucleosomal DNA and nucleosomal DNA (Figure 6a), indicating that DNA compaction in the NCP does not alter the Watson–Crick base pairing dynamics or stability. Additionally, in agreement with the cryo-EM structures, the global NCP dynamics were not impacted by the presence of an 8oxoG:C base pair. However, local perturbations of the DNA backbone were observed at the site of the 8oxoG:C base pair. In each 8oxoG:C-NCP system, the distance between O8 of 8oxoG and the closest non-bridging oxygen atom was greater than that for the H8 atom of G and the closest non-bridging oxygen in the ND-NCP system (Figure 6b,c), reflecting altered local DNA backbone geometry due to the presence of 8oxoG:C. Importantly, this local rearrangement of the DNA backbone is observed in both the 8oxoG:C-NCP systems and the duplex DNA system. Finally, we were interested in testing whether the increased amount of bridging water molecules and sodium cations present in the 8oxoG:C system compared to the ND-NCP systems impacted the DNA backbone angle distribution (Figures S12 and S13). We observed that the non-damaged systems exhibit a single distribution of α and γ backbone angles, whereas the α and γ dihedral angles of the 8oxoG nucleotide exhibit a position-dependent bimodal distribution for 8oxoG:C (Figure 6d). This bimodality is especially pronounced at SHL+4 which may be a result of contacts made between the 8oxoG:C base pair and the histone H2A tail at this site (Figure 4c). Importantly, this bimodality is also observed in the duplex DNA system (Figure 6d). Cumulatively, these data indicate that the presence of 8oxoG destabilizes the phosphate backbone, but this destabilization is largely unchanged between nucleosomal and non-nucleosomal DNA. Overall, the intrinsic structure and dynamics of 8oxoG:C and 8oxoG:A base pairs are unchanged between nucleosomal and non-nucleosomal DNA, suggesting that lesion presentation does not influence whether lesion excision can occur.

## 4. Discussion

Oxidative damage of the nucleobase G gives rise to the mutagenic DNA lesion 8oxoG, which occurs across the genome in chromatinized and non-chromatinized DNA. How 8oxoG is accommodated within the NCP and how oxidative DNA glycosylases repair 8oxoG lesions in chromatin remains poorly understood. Using a combination of biochemical assays, structural biology, and classical MD simulations, we characterized the structure and dynamics of 8oxoG:C and 8oxoG:A base pairs in the NCP. In addition, we established that MUTYH exhibits negligible activity in repairing 8oxoG:A base pairs in the NCP, despite the comparable conformation of the 8oxoG:A base pair in nucleosomal DNA relative to non-nucleosomal DNA. Together, these findings establish that while 8oxoG is readily accommodated within nucleosomal DNA, MUTYH is effectively excluded from the NCP at all regions besides the entry/exit site in our experiments reported here. Importantly, this work provides a structural and biochemical basis for the elevated G to T transversion rate associated with oxidative DNA damage in chromatin [6,27].

Prior work established that in non-nucleosomal DNA, 8oxoG adopts the *anti* conformation and utilizes Watson–Crick base pairing opposite C, whereas 8oxoG adopts the *syn* conformation and utilizes Hoogsteen base pairing opposite A [70,72]. Recent structures of 8oxoG in the NCP revealed that 8oxoG uses Watson–Crick base pairing with C [6,40,41]. However, little is known about the base pairing properties of 8oxoG:A in the NCP, and whether these properties are position dependent. The cryo-EM structures shown here reveal that nucleosomal 8oxoG will base pair with C and A using Watson–Crick and Hoogsteen base pairing, respectively, similar to what was observed in non-nucleosomal DNA. Importantly, these base pairing properties remain nearly identical across the four translational positions we tested, indicating that the ability of 8oxoG to adopt *anti* or *syn* conformations is not dependent on the local DNA and/or histone environment. Furthermore, we found that the presence of the 8oxoG lesion in the NCP did not cause major perturbations to the histone octamer and induced only very local structural perturbations to the DNA backbone. The MD simulations reveal that in the local DNA backbone, there is an equilibrium between two backbone conformations for 8oxoG:C and 8oxoG:A in both nucleosomal and non-nucleosomal DNA. Additionally, there is a specific dynamic behavior of the nucleobases in the 8oxoG:A base pair that was not observed for the 8oxoG:C base pair. Together, these data indicate that 8oxoG is readily accommodated in the NCP, though the 8oxoG:A base pair is more dynamic compared to the 8oxoG:C base pair. The ability of the NCP to accommodate 8oxoG suggests that the presence of 8oxoG also likely has minimal impact on nucleosome assembly and/or disassembly.

The primary mechanism for repairing 8oxoG lesions within chromatinized and non-chromatinized DNA is the BER pathway. The method by which BER is initiated to repair 8oxoG is dependent upon its base pairing partner, where repair of 8oxoG:C is initiated by OGG1, and repair of 8oxoG:A is initiated by MUTYH [17]. Prior work identified that OGG1 excises 8oxoG in the NCP, though with reduced efficiency compared to non-nucleosomal DNA, suggesting that 8oxoG:C can be repaired in the context of chromatin [31,32]. The reduced efficiency of OGG1 is dictated by the position of the 8oxoG:C in the NCP, where solution accessible 8oxoG is excised more readily than histone-occluded 8oxoG. In contrast to OGG1, our data indicate that MUTYH has minimal activity in the NCP regardless of the rotational orientation of 8oxoG, though activity was observed at the NCP entry/exit site. Notably, our cryo-EM data collection efforts to determine structures of MUTYH bound to NCPs containing 8oxoG:A were unsuccessful across multiple lesion positions, consistent with the biochemical evidence for MUTYH exclusion from the NCP. Structure-activity relationships reveal that MUTYH relies on the 2-amino group of 8oxoG for detection of the 8oxoG:A base pair and utilizes two domains to engage with both the 8oxoG and A containing strands to form a catalytically active complex on non-nucleosomal DNA [47]. Specifically, a conserved C-terminal domain β-FSH loop invades the DNA helix and aids in 8oxoG recognition, while the complementary A is accommodated into the active site within the N-terminal domain [18,21,22,73]. While the placement of the 8oxoG base pair in our structures features the 2-amino group of 8oxoG toward the histone octamer, our biochemical data suggest that there is no significant A excision by MUTYH regardless of the orientation of the 2-amino group in our conditions and instead is dependent on the lesion being present at the entry/exit site. Structural modeling using available MUTYH structures indicates that within an NCP, MUTYH cannot form the extensive DNA contacts that are required to engage an 8oxoG:A lesion without significant clashing with the core histones or nucleosomal DNA (Figure S18), providing a structural explanation for its exclusion. The activity observed at the entry/exit site is consistent with the dynamic nature of DNA at this position; when the terminal DNA transiently dissociates from the histone octamer, MUTYH can encompass the lesion in a catalytically competent state and excise A.

Failure to repair an 8oxoG:A base pair will result in a G to T transversion which is a common mutational signature in cancer [13] and is associated with dysfunctional MUTYH variants in MAP (SBS 36) [74]. Prior work showed that G to T transversions occur at a higher rate in histone bound DNA compared to linker DNA between NCPs following oxidative stress in human cells [6,27]. These observations are consistent with the role of MUTYH as a post-replicative repair enzyme [75], rather than a housekeeping enzyme that constitutively surveys the genome for 8oxoG:A lesions. 8oxoG:A base pairs arise primarily when replicative polymerases misincorporate A opposite 8oxoG during DNA synthesis, at which point the DNA is transiently free of histones. MUTYH associates with PCNA and is recruited to replication forks, providing a window to excise the misincorporated A before newly synthesized DNA is repackaged into chromatin [76,77]. While the inability of MUTYH to access 8oxoG:A lesions in nucleosomal DNA could, in principle, contribute to GC content erosion in chromatin-packaged genomic regions over evolutionary timescales, this risk is mitigated by the post-replicative timing of MUTYH activity. Any 8oxoG:A base pairs that escape this post-replicative repair window and are packaged into nucleosomes would be largely inaccessible to MUTYH, impacting the G to T transversion rate observed in histone-bound DNA.

The inability of MUTYH to function at most sites in the NCP provides a strong rationale for the observed mutational spectra. However, in vitro activity of BER enzymes does not correlate with the magnitude of mutational frequency, suggesting that additional cellular factors, including chromatin remodelers and BER cofactors, which have been shown to stimulate BER activity in NCPs, likely help facilitate the repair of oxidative DNA damage in chromatin [78,79,80,81]. Chromatin remodeling could promote repositioning of a nucleosomal 8oxoG:A base pair to the linker region between nucleosomes that is more accessible to BER proteins. Several chromatin remodelers have been shown to be epistatic with BER factors including Amplified in Liver Cancer 1 (ALC1) [82], Chromodomain Helicase DNA-binding protein 6 (CHD6) [83], and HELLS [84]. Additionally, other BER cofactors could help promote MUTYH activity in vivo that is not observed in our in vitro experiments. One cofactor that has been shown to be rapidly recruited to oxidative damage within chromatin and stimulate both OGG1 and MUTYH activity is the DNA damage binding protein, UV-DDB [85,86]. It is possible that adding UV-DDB to our experiments could increase MUTYH activity. Lastly, the data presented here does not exclude the possibility that MUTYH could function in other chromatinized settings, including hexasomes or tetrasomes where the DNA is more dynamic and perhaps the 2-amino group is more accessible to facilitate 8oxoG:A recognition by MUTYH [87,88]. Future studies will be required to dissect the contribution of lesion positioning, DNA sequence, chromatin states, chromatin remodelers, and BER cofactors to MUTYH-initiated BER.

## 5. Conclusions

The work presented here establishes that 8oxoG:A base pairs are structurally accommodated within the nucleosome core particle, adopting Hoogsteen geometry identical to that observed in non-nucleosomal DNA across four distinct superhelical locations. MD simulations further reveal that the dynamic behavior of 8oxoG:A observed in non-nucleosomal DNA, including a two-state conformational exchange of the nucleobase and localized DNA backbone perturbations, is preserved in the nucleosome context. Despite this structural accommodation, MUTYH is effectively excluded from the NCP, with adenine excision activity limited to the entry/exit region of the nucleosome in our experimental approach. Collectively, these findings demonstrate that steric exclusion by the histone octamer, rather than altered lesion presentation, is the primary barrier to MUTYH-initiated repair of 8oxoG:A in chromatin.

These results provide a structural and biochemical rationale for the elevated G to T transversion rate observed in histone-bound DNA following oxidative stress. While post-replicative recruitment of MUTYH to replication forks provides a window for repair before newly synthesized DNA is repackaged into nucleosomes, 8oxoG:A base pairs that escape this window are largely inaccessible to MUTYH within the NCP. The current study uses the Widom 601 strong positioning sequence with 8oxoG:A systematically introduced at four superhelical locations, but it remains to be determined whether different DNA sequences, additional translational positions, or alternative lesion base pair combinations alter the extent of MUTYH exclusion from the nucleosome. Future work will also be required to determine how chromatin remodeling enzymes, BER cofactors such as UV-DDB, and alternative chromatinized states influence MUTYH-initiated repair in the broader context of chromatin.

## Data Availability

Atomic coordinates for the reported structures have been deposited to the Protein Data Bank under accession numbers 10GJ, 10GK, 10GL, 10GM, 10GN, and 10GO. Cryo-EM maps are available from the Electron Microscopy Data Bank under accession numbers EMD-75152, EMD-75153, EMD-75154, EMD-75155, EMD-75156, and EMD-75157. Atomic coordinates for the initial nucleosome model were obtained from the Protein Data Bank under accession number 7U51. All MD data is available online on a Zenodo repository (https://doi.org/10.5281/zenodo.14755037).

## Supplementary Materials

The following supporting information can be downloaded at: https://www.mdpi.com/article/10.3390/biom16070999/s1, Supplementary Materials S1: Figure S1: Nucleosome assembly for enzyme kinetics; Figure S2: Biochemical analysis of A excision via MUTYH; Figure S3: Nucleosome reconstitution for cryo-EM studies; Figure S4: Structural determination of the 8oxoG:A base pair at multiple translational positions in the nucleosome; Figure S5: SPA processing workflow for 8oxoG:A-NCP+2 and 8oxoG:A-NCP+3; Figure S6: SPA processing workflow for 8oxoG:A-NCP+4 and 8oxoG:A-NCP−6; Figure S7: 8oxoG:A-NCP+2 map and model quality assessment; Figure S8: 8oxoG:A-NCP+3 map and model quality assessment; Figure S9: 8oxoG:A-NCP+4 map and model quality assessment; Figure S10: 8oxoG:A-NCP−6 map and model quality assessment; Figure S11: Intra-base pair parameters 5’ of the damage site; Figure S12: Solvation shell around the 8oxoG O8 atom; Figure S13: Na^+^ cations distribution around the 8oxoG O8 atom; Figure S14: Structural detertmination of the 8oxoG:C base pair at multiple translational positions in the nucleosome; Figure S15: SPA processing workflow for 8oxoG:C-NCP+2 and 8oxoG:C-NCP+3; Figure S16: 8oxoG:A-NCP+2 map and model quality assessment; Figure S17: 8oxoG:C-NCP+3 map and model quality assessment; Figure S18: Modeled MUTYH recognition mechanism in the NCP; Table S1: Solvent exposure and MUTYH excision activity on dA sites; Table S2: Cryo-EM table for 8oxoG:A and 8oxoG:C NCPs; Table S3: DNA oligonucleotides used for reconstitution of NCPs for cryo-EM studies. Supplementary Materials S2: The original images of Supplementary Materials S1. Supplementary Materials S3: video.
