# Supplementary material for: 8oxoG:A Is Structurally Accommodated in the Nucleosome Core Particle, Yet Inaccessible to MUTYH-Initiated DNA Repair
Source: Biomolecules. 2026 Jul 8;16(7):999. doi: 10.3390/biom16070999 (PMC13406648; doi:10.3390/biom16070999)
Supplement: Supplementary file 1 [file biomolecules-16-00999-s001.zip › Supplementary Materials S2. Updated-8oxoGA_rawimages.pdf]

Native gels corresponding to Supplementary Fig 3

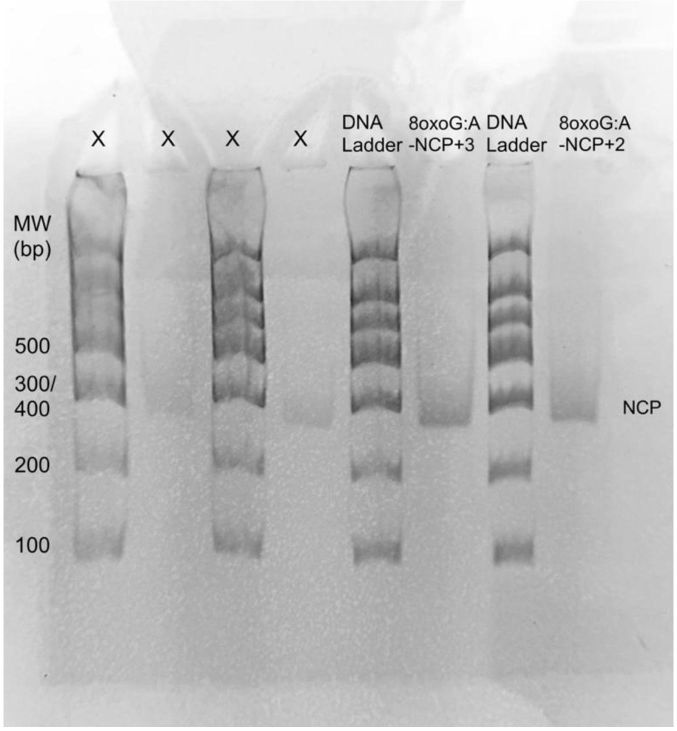

Native gel showing nucleosome reconstitution for 8oxoG:A-NCP+3 and 8oxoG:A-NCP+2.

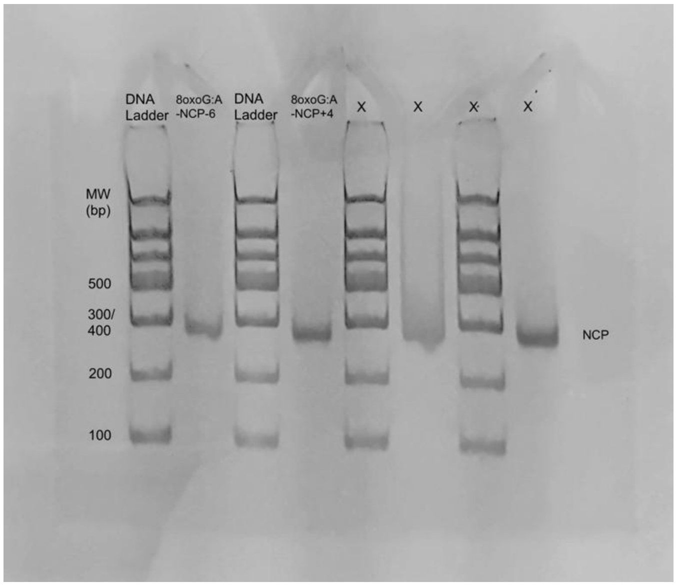

Native gel showing nucleosome reconstitution for 8oxoG:A-NCP-6 and 8oxoG:A-NCP+4.

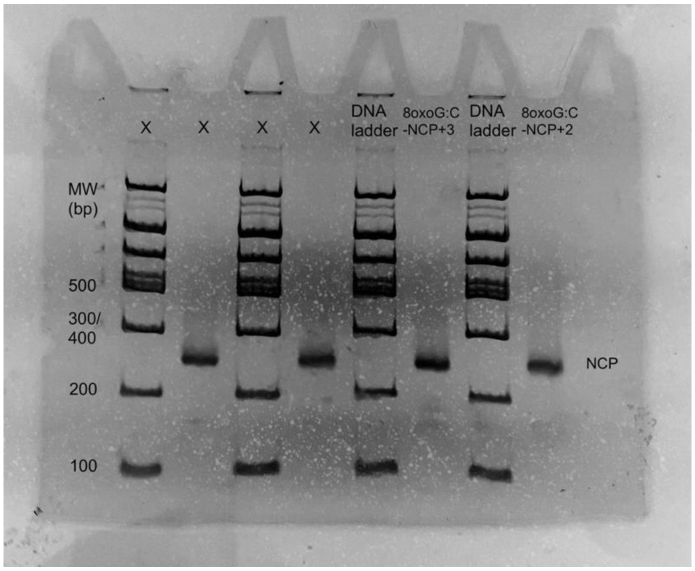

Native gel showing nucleosome reconstitution for 8oxoG:C-NCP+3 and 8oxoG:C-NCP+2

Native gels corresponding to Supplementary Fig 5

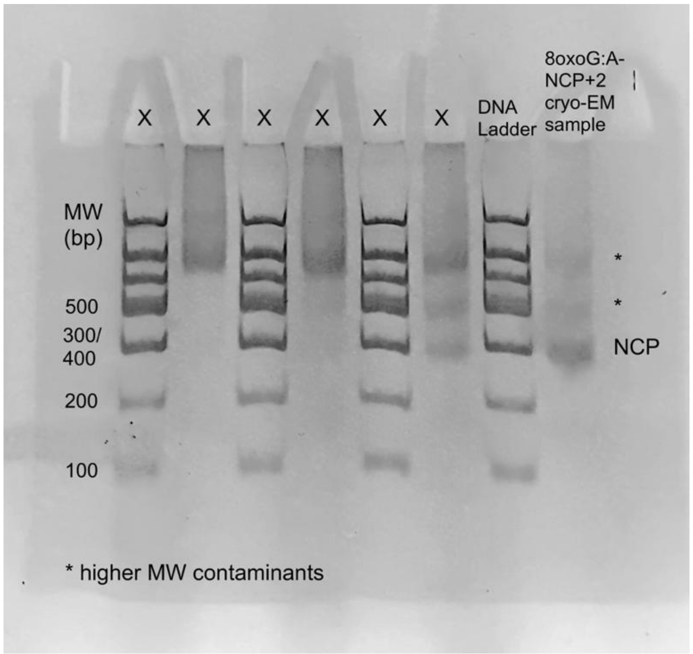

Native gel showing the cryo-EM sample for the 8oxoG:A-NCP+2 structure.

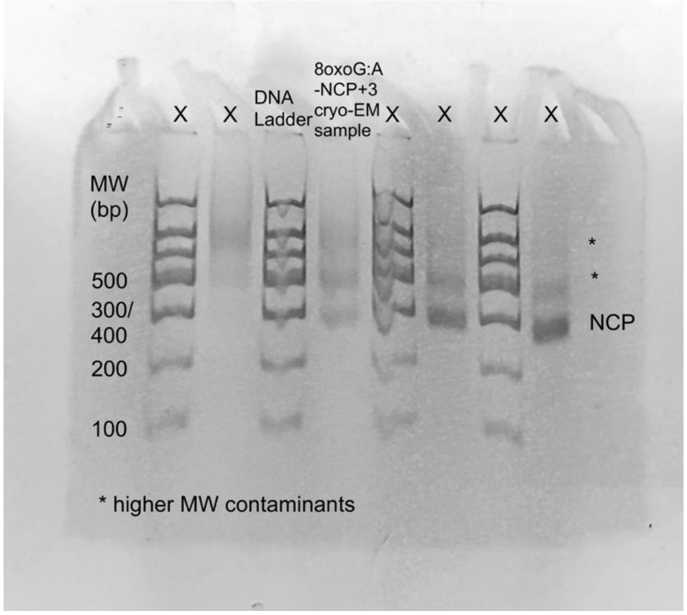

Native gel showing the cryo-EM sample for the 8oxoG:A-NCP+3 structure.

Native gels corresponding to Supplementary Fig 6

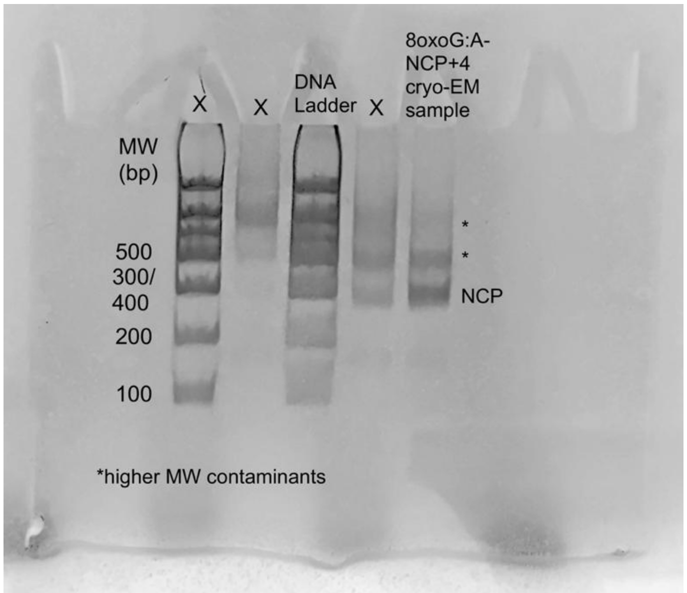

Native gel showing the cryo-EM sample for the 8oxoG:A-NCP+4 structure.

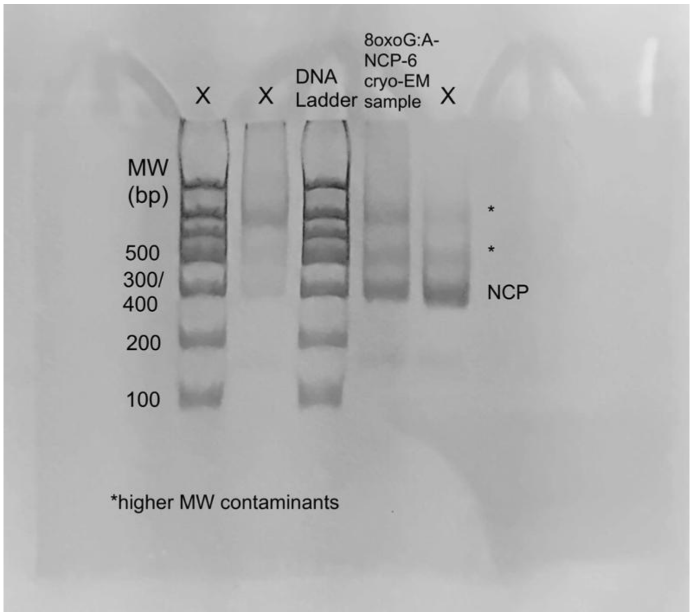

Native gel showing the cryo-EM sample for the 8oxoG:A-NCP-6 structure.

Native gels for Supplementary Fig 15

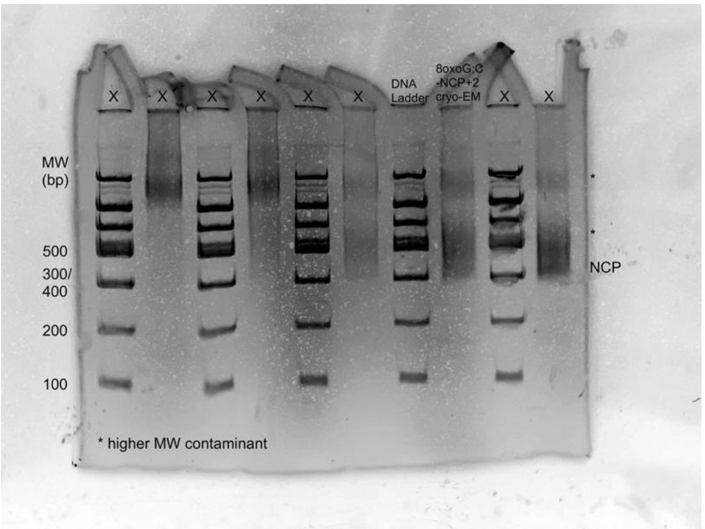

Native gel showing the cryo-EM sample for the 8oxoG:C-NCP+2 structure.

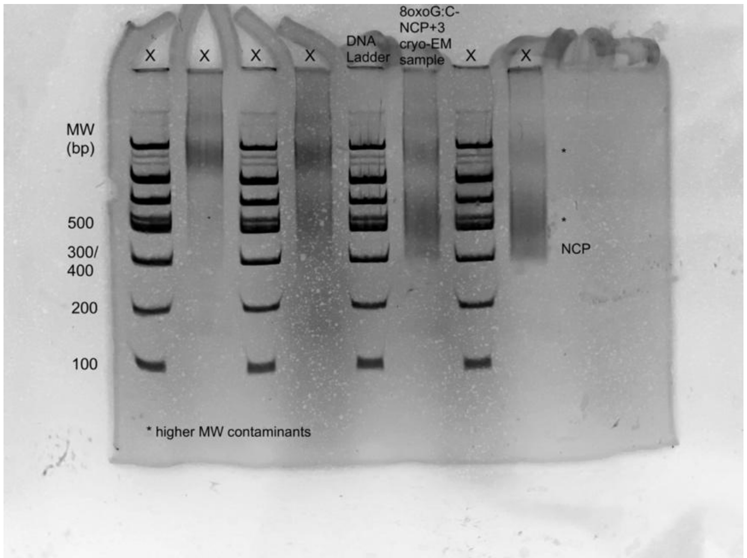

Native gel showing the cryo-EM sample for the 8oxoG:C-NCP+3 structure.
