# Supplementary material for: 8oxoG:A Is Structurally Accommodated in the Nucleosome Core Particle, Yet Inaccessible to MUTYH-Initiated DNA Repair
Source: Biomolecules. 2026 Jul 8;16(7):999. doi: 10.3390/biom16070999 (PMC13406648; doi:10.3390/biom16070999)
Supplement: Supplementary file 1 [file biomolecules-16-00999-s001.zip › Supplementary Materials S1. biomolecules-supplementary-R1_BF.pdf]

**SUPPLEMENTAL MATERIAL**

for

# 8oxoG:A is structurally accommodated in the nucleosome core particle, yet inaccessible to MUTYH initiated DNA repair

Abigayle F. Vito[1*], Justin A. Ling[1*], Julia C. Ferrara[2], Caleb S. Jacques[2], Natacha Gillet[3], Roy González-Alemán[4], Yuya Qiu[4], Mohammad Hashemian[5], Carlos H. Trasviña-Arenas[5], Sheila S. David[5], Sarah Delaney[2], Emmanuelle Bignon[4#], Bret D. Freudenthal[1,6,7#]

[1] Department of Biochemistry and Molecular Biology, University of Kansas Medical Center, Kansas City, Kansas, 66160, USA.

[2] Department of Chemistry, Brown University, Providence, Rhode Island, 02912, USA.

[3] CNRS, ENS de Lyon, Laboratoire de Chimie UMR 5182, 46 allée d'Italie, 69364 Lyon, France

[4] Université de Lorraine, CNRS, UMR 7019, LPCT, F-54000 Nancy, France

[5] Department of Chemistry, University of California, Davis, California, 95616, USA.

[6] Department of Cancer Biology, University of Kansas Medical Center, Kansas City, Kansas, 66160, USA.

[7] University of Kansas Cancer Center, Kansas City, KS, 66160, USA.

* These authors contributed equally to this work.

[#] To whom correspondence should be addressed: Tel: +1 913 588 5560; Fax: +1 913 588 9896;

Emails: Emmanuelle Bignon (emmanuelle.bignon@univ-lorraine.fr), and Bret D. Freudenthal (bfreudenthal@kumc.edu)

**Table of Contents**

**Figure S1.** Nucleosome assembly for enzyme kinetics ……………….………………….…..……S3

**Figure S2.** Biochemical analysis of dA excision via MUTYH ….…………………………….…S4

**Figure S3.** Nucleosome reconstitution for cryo-EM studies……………..………….……..……..S5

**Figure S4.** Structural determination of the 8oxoG:A base pair at multiple translational positions in the nucleosome….……………………………………………………………………….……..S6

**Figure S5.** SPA processing workflow for 8oxoG:A-NCP+2 and 8oxoG:A-NCP+3…..…………S7-8

**Figure S6.** SPA processing workflow for 8oxoG:A-NCP+4 and 8oxoG:A-NCP−6……..……S9-10

**Figure S7.** 8oxoG:A-NCP+2 map and model quality assessment.…………………...……S11-12

**Figure S8.** 8oxoG:A-NCP+3 map and model quality assessment.…………………………S13-14

**Figure S9.** 8oxoG:A-NCP+4 map and model quality assessment.……………………….…S15-16

**Figure S10.** 8oxoG:A-NCP−6 map and model quality assessment.….……………..…..…S17-18

**Figure S11.** Intra-base pair parameters 5′ of the damage site……………………..………...…S19

**Figure S12.** Solvation shell around the 8oxoG O8 atom.……………………………………...…S20

**Figure S13** $Na^+$ cations distribution around the 8oxoG O8 atom.…….…………….………….…S21

**Video S1.** Dynamics of the 8oxoG backbone in the 8oxoG:A mismatch.................................S22

**Figure S14.** Structural determination of the 8oxoG:C base pair at multiple translational positions in the nucleosome….………………………………………………………………..……..S23

**Figure S15.** SPA processing workflow for 8oxoG:C-NCP+2 and 8oxoG:A-NCP+3…..…..…S24-25

**Figure S16.** 8oxoG:C-NCP+2 map and model quality assessment……………….......……S26-27

**Figure S17.** 8oxoG:C-NCP+3 map and model quality assessment…………….……….…S28-29

**Figure S18.** Modeled MUTYH recognition mechanism in the NCP…………….………….…..S30

**Table S1.** Solvent Exposure and MUTYH Excision Activity on dA Sites................................S31

**Table S2.** Cryo-EM table for 8oxoG:A and 8oxoG:C NCPs................................................S32-33

**Table S3.** DNA oligonucleotides used for reconstitution of NCPs for cryo-EM studies.......S34-35

**Supplementary Figure S1**

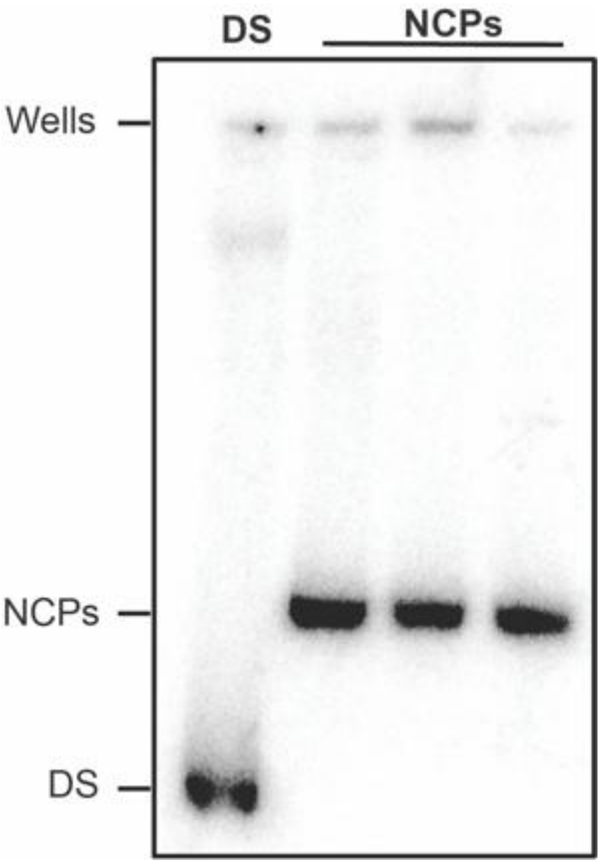


**Supplementary Figure S1: Nucleosome assembly for enzyme kinetics studies**
Representative native PAGE analyzing NCP assembly. The double stranded DNA control is labeled DS. The three NCP lanes reflect migration of the NCP samples relative to control.

**Supplementary Figure S2**

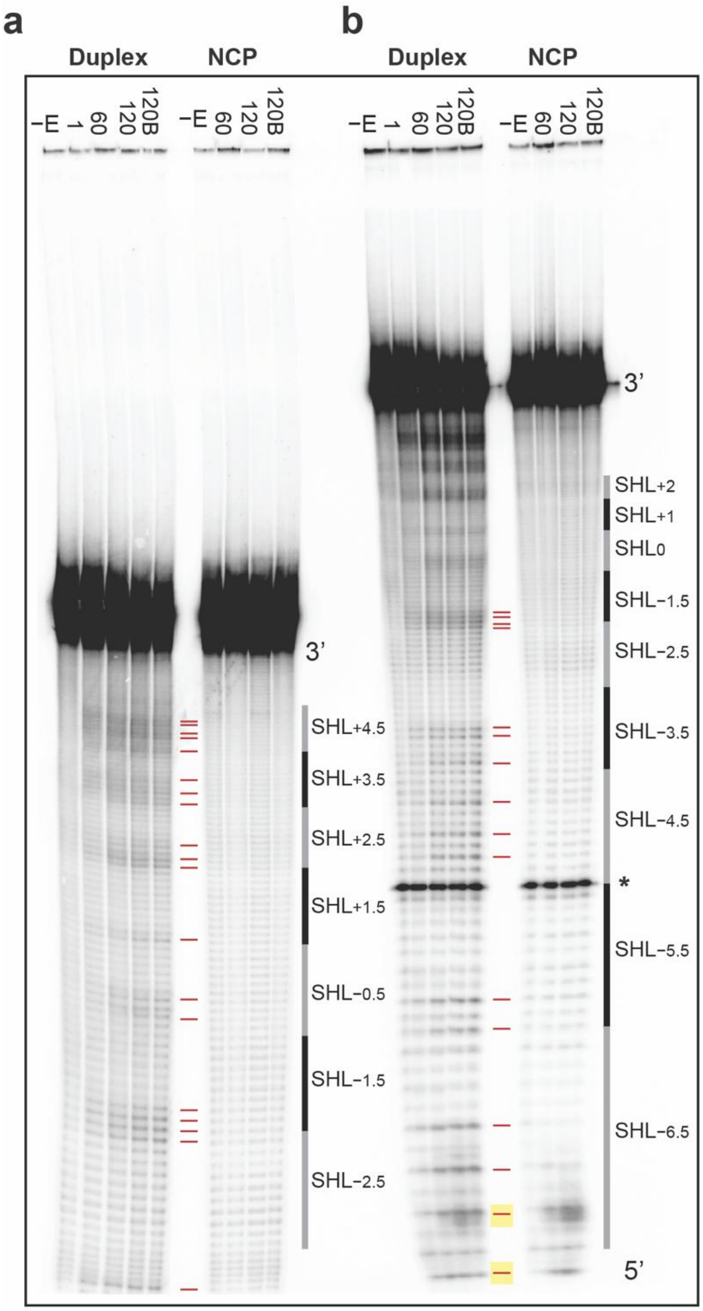


**Supplementary Figure S2: Biochemical analysis of A excision via MUTYH**

**a,** Representative 10 % denaturing PAGE showing A excision from 8oxoG:A in non-nucleosomal (duplex) and nucleosomal DNA (NCP) by MUTYH. −E samples are substrate (duplex or NCP) incubated for 120 min without MUTYH to reveal background damage that was pre-existing or occurred through sample workup. Substrates (duplex or NCP) incubated with MUTYH are labeled by their incubation time: 1, 60, or 120 min. The 120B sample was incubated with MUTYH for 60 min, supplemented with additional MUTYH, and incubated for an additional 60 min. Red dashes mark locations of dA. The asterisk indicates a loading standard. SHL of the NCP are indicated. Gel was run for 3 h to visualize SHL−2 to SHL+4. **b,** Same as (a) except the gel was run for 1.5 h to visualize SHL−6.5 to SHL−3. A that were excised from NCPs by MUTYH are highlighted in yellow.

**Supplementary Figure S3**

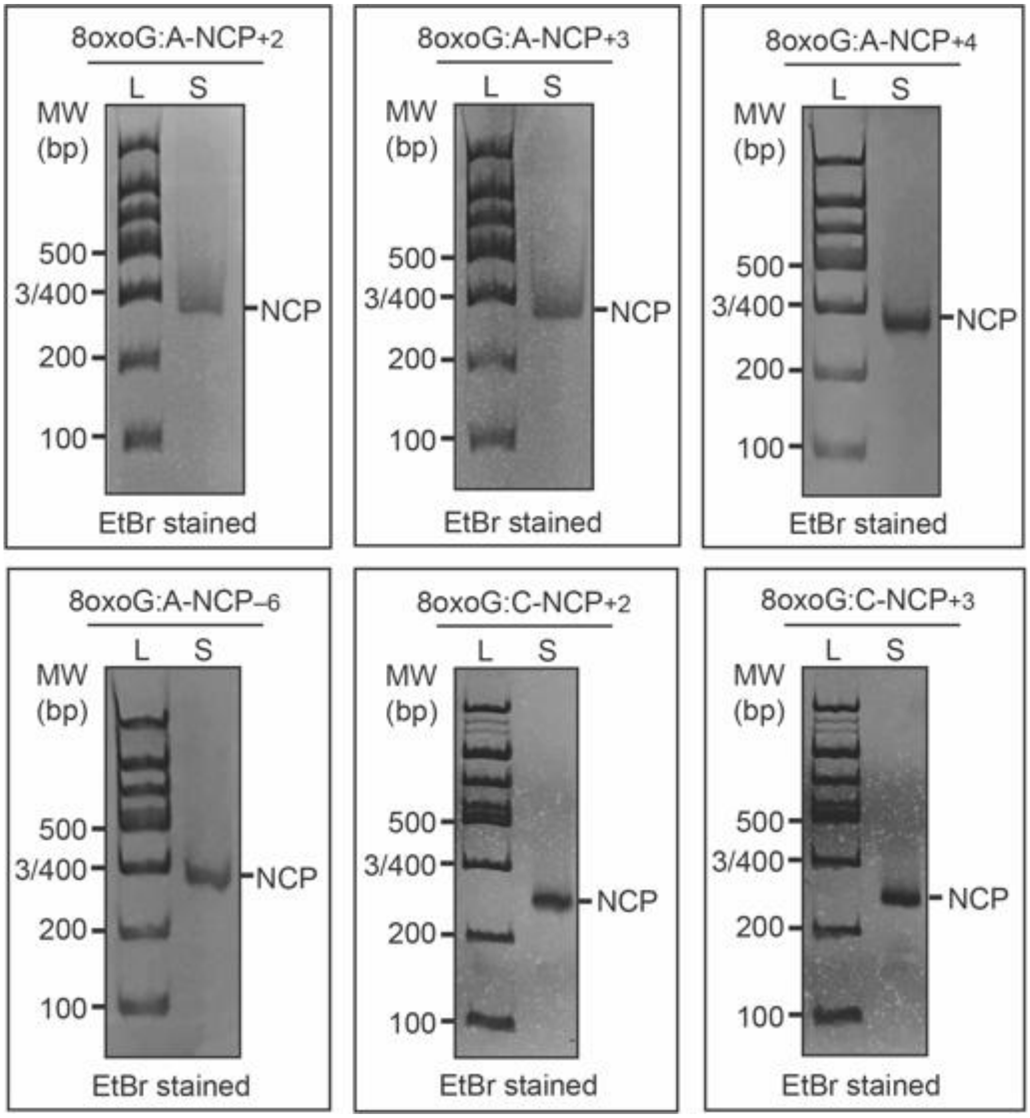


**Supplementary Figure S3: Nucleosome reconstitution for cryo-EM studies**
Native PAGE gels confirming nucleosome formation and purity for the 8oxoG:C-NCP+2, ND-NCP, 8oxoG:C-NCP+3, 8oxoG:A-NCP+2, 8oxoG:A-NCP+4, and 8oxoG:A-NCP–6. The native PAGE gels were run immediately after initial nucleosome formation and purification. The NCPs were detected using ethidium bromide staining. The 100 bp DNA ladder (L) and nucleosome sample (S) are labeled.

**Supplementary Figure S4**

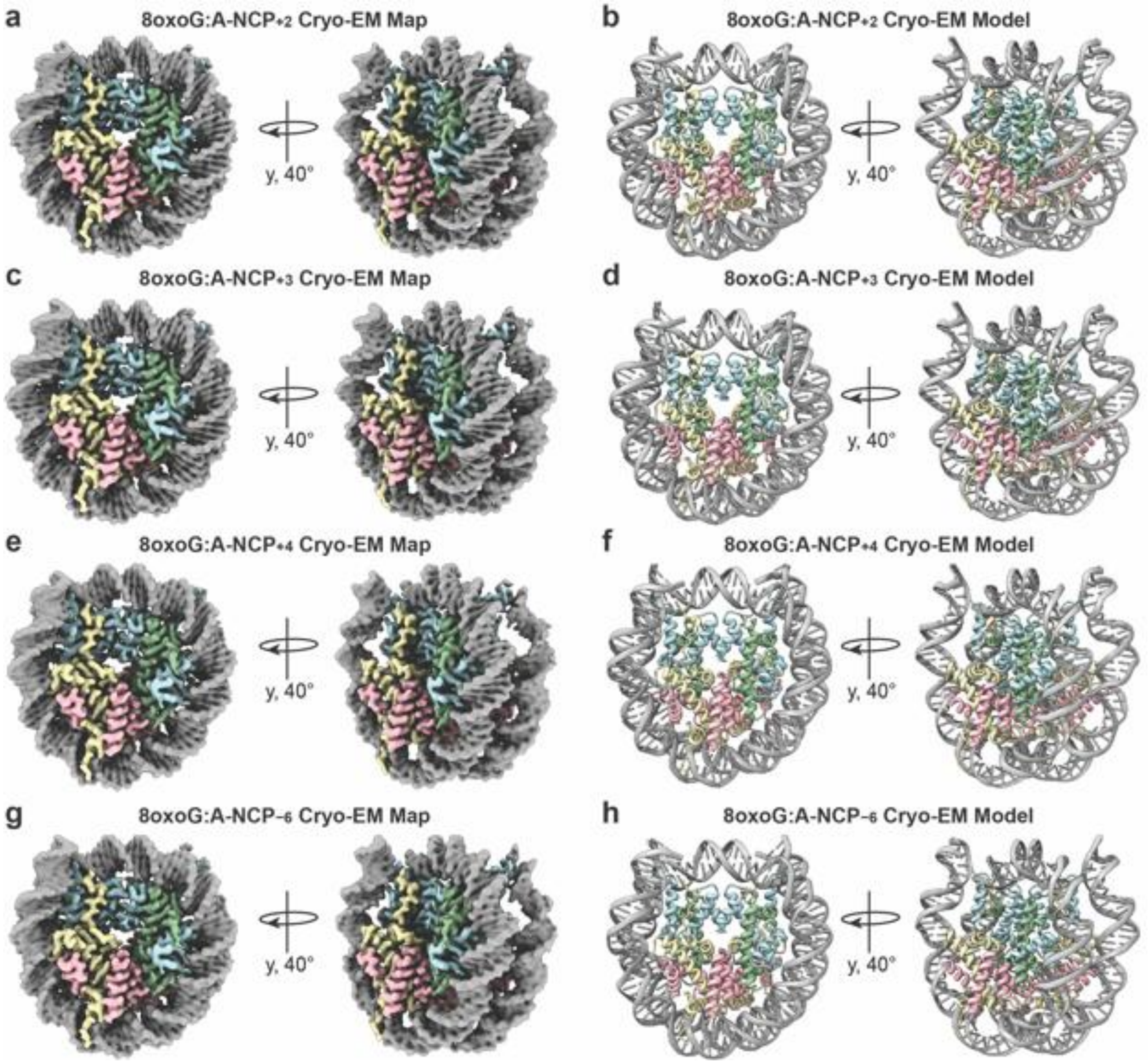


**Supplementary Figure S4: Structural determination of the 8oxoG:A base pair at multiple translational positions in the nucleosome**

Final 8oxoG:A-NCP cryo-EM map and model shown in two orientations at SHL+2 (**a,b**), SHL+3 (**c,d**), SHL+4 (**e,f**), and SHL−6 (**g,h**).

**Supplementary Figure S5**

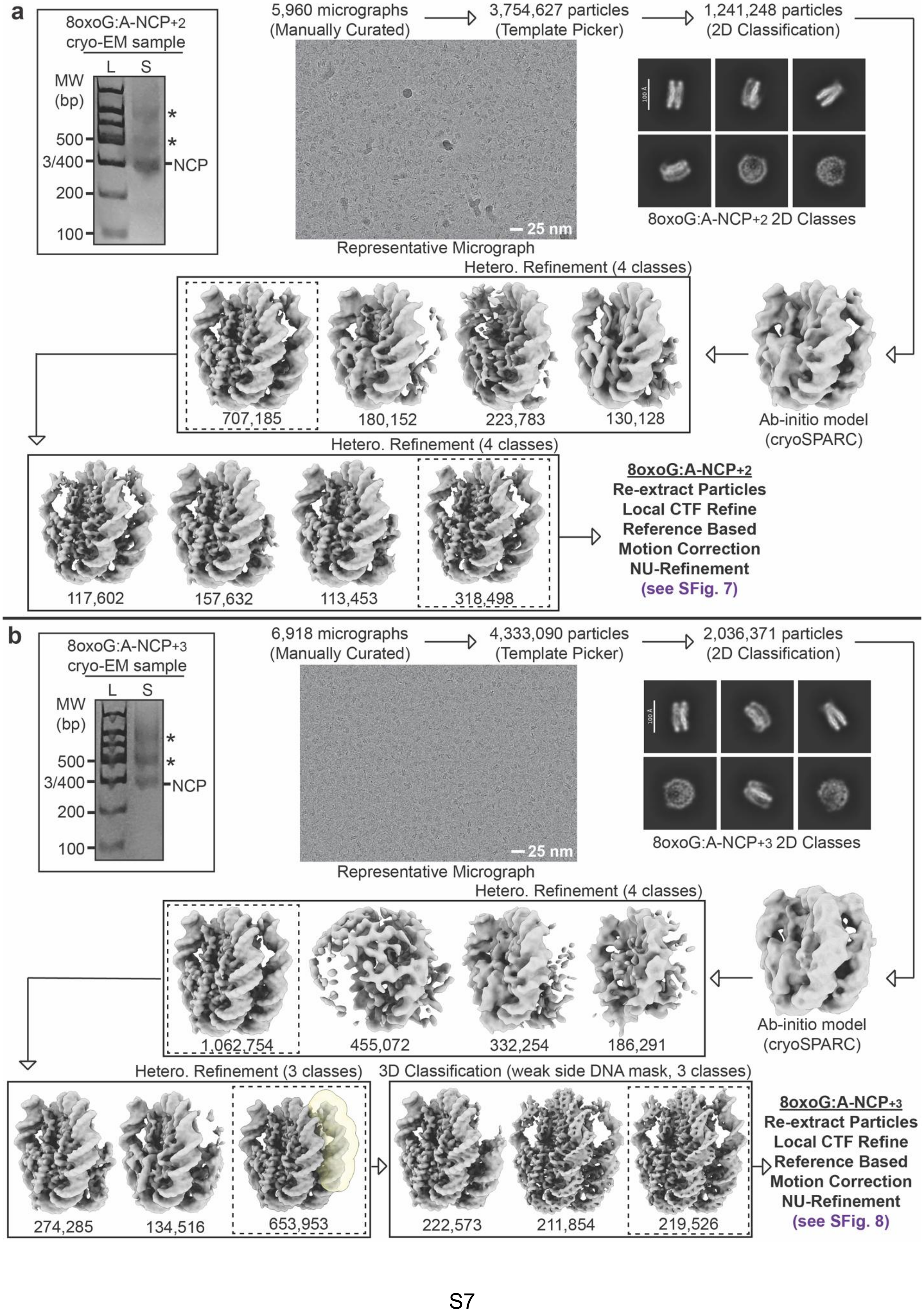

**Supplementary Figure S5: SPA processing workflow for 8oxoG:A-NCP+2 and 8oxoG:A-NCP+3**

**a,** Native PAGE gel of 8oxoG:A-NCP+2 cryo-EM sample and flowchart of the data processing pipeline for the 8oxoG:A-NCP+2 cryo-EM dataset. A 100 bp DNA ladder (L) and the cryo-EM sample (S) are labeled and the NCP was detected using ethidium bromide staining. * indicates higher MW contaminants. A representative micrograph and representative 2D classes from the 8oxoG:A-NCP+2 cryo-EM dataset are shown. The final maps, final models, and quality assessment metrics for 8oxoG:A-NCP+2 can be found in Supplementary Fig. 7. **b,** Native PAGE gel of 8oxoG:A-NCP+3 cryo-EM sample and flowchart of the data processing pipeline for the 8oxoG:A-NCP+3 cryo-EM dataset. A 100 bp DNA ladder (L) and the cryo-EM sample (S) are labeled and the NCP was detected using ethidium bromide staining. * indicates higher MW contaminants. A representative micrograph and representative 2D classes from the 8oxoG:A-NCP+3 cryo-EM dataset are shown. The DNA focus mask used for 3D classification is shown in a transparent yellow surface. The final maps, final models, and quality assessment metrics for 8oxoG:A-NCP+3 can be found in Supplementary Fig. 8.

## Supplementary Figure S6

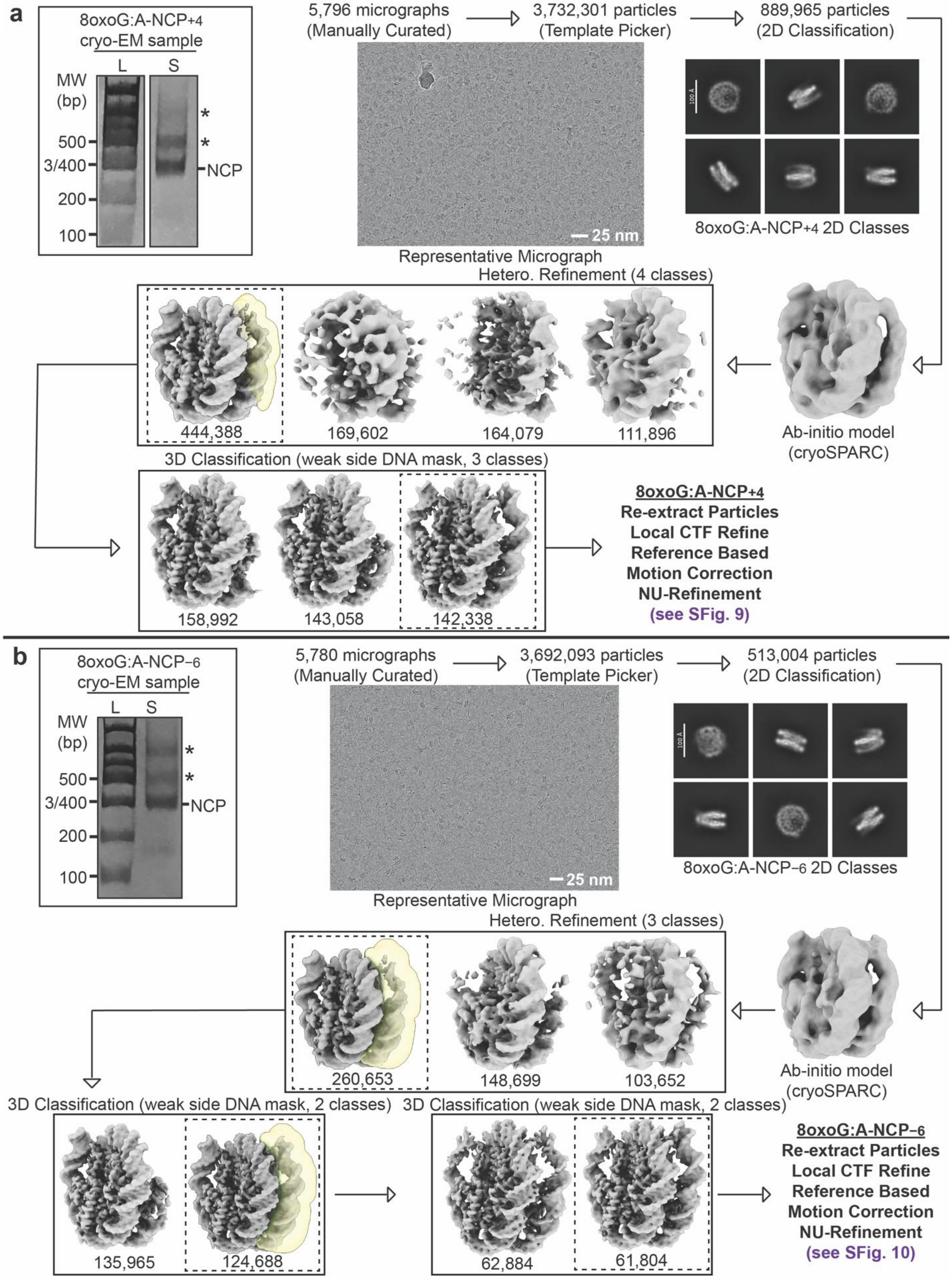

**Supplementary Figure S6: SPA processing workflow for 8oxoG:A-NCP$_{+4}$ and 8oxoG:A-NCP$_{-6}$**

**a,** Native PAGE gel of 8oxoG:A-NCP$_{+4}$ cryo-EM sample and flowchart of the data processing pipeline for the 8oxoG:A-NCP$_{+4}$ cryo-EM dataset. A 100 bp DNA ladder (L) and the cryo-EM sample (S) are labeled and the NCP was detected using ethidium bromide staining. * indicates higher MW contaminants. A representative micrograph and representative 2D classes from the 8oxoG:A-NCP$_{+4}$ cryo-EM dataset are shown. The DNA focus mask used for 3D classification is shown in a transparent yellow surface. The final maps, final models, and quality assessment metrics for 8oxoG:A-NCP$_{+4}$ can be found in Supplementary Fig. 9. **b,** Native PAGE gel of 8oxoG:A-NCP$_{-6}$ cryo-EM sample and flowchart of the data processing pipeline for the 8oxoG:A-NCP$_{-6}$ cryo-EM dataset. A 100 bp DNA ladder (L) and the cryo-EM sample (S) are labeled and the NCP was detected using ethidium bromide staining. * indicates higher MW contaminants. A representative micrograph and representative 2D classes from the 8oxoG:A-NCP$_{-6}$ cryo-EM dataset are shown. The DNA focus mask used for 3D classification is shown in a transparent yellow surface. The final maps, final models, and quality assessment metrics for 8oxoG:A-NCP$_{-6}$ can be found in Supplementary Fig. 10.

**Supplementary Figure S7**

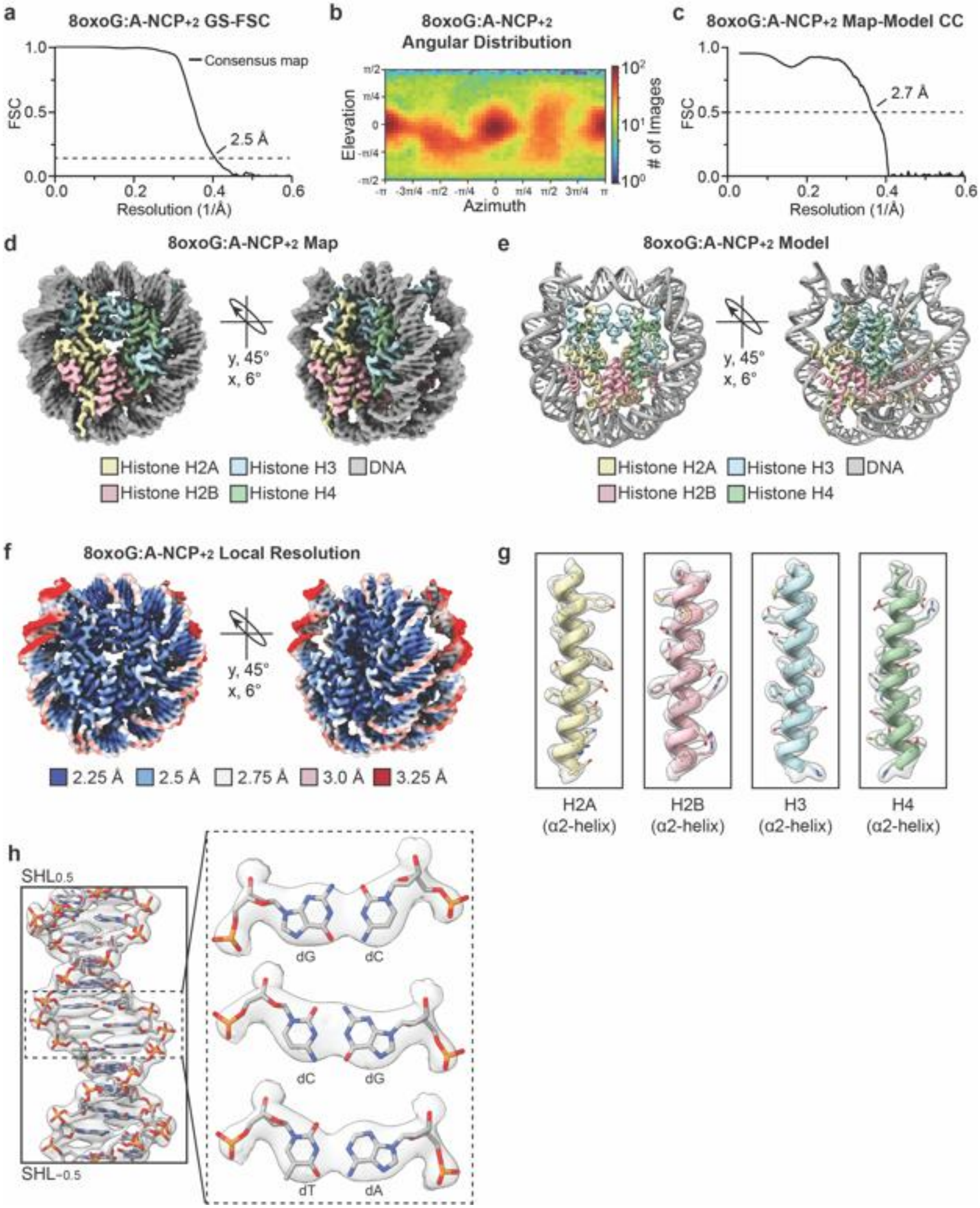

**Supplementary Figure S7: 8oxoG:A-NCP+2 map and model quality assessment**
**a,** Gold-standard Fourier shell correlation (GS-FSC) curve for the 8oxoG:A-NCP+2 cryo-EM map (black solid line). The dashed line corresponds to FSC – 0.143. **b,** Angular distribution heatmap for the 8oxoG:A-NCP+2 cryo-EM map. **c,** Map-to-model FSC curve for the 8oxoG:A-NCP+2 model and 8oxoG:A-NCP+2 cryo-EM map. The dashed line corresponds to FSC – 0.5. **d,** The final 2.5 Å 8oxoG:A-NCP+2 cryo-EM map shown in two orientations. **e,** The final 8oxoG:A-NCP+2 model shown in two orientations. **f,** The local resolution estimation for the 8oxoG:A-NCP+2 cryo-EM map shown in two orientations. **g,** Representative segmented densities for histones H2A, H2B, H3, and H4 in the 8oxoG:A-NCP+2 cryo-EM map. The representative segmented densities from the cryo-EM map are shown as transparent gray surfaces. **h,** DNA base pair densities for base pairs at the dyad in the 8oxoG:A-NCP+2 cryo-EM map. The segmented densities from the cryo-EM map are shown as transparent gray surfaces.

**Supplementary Figure S8**

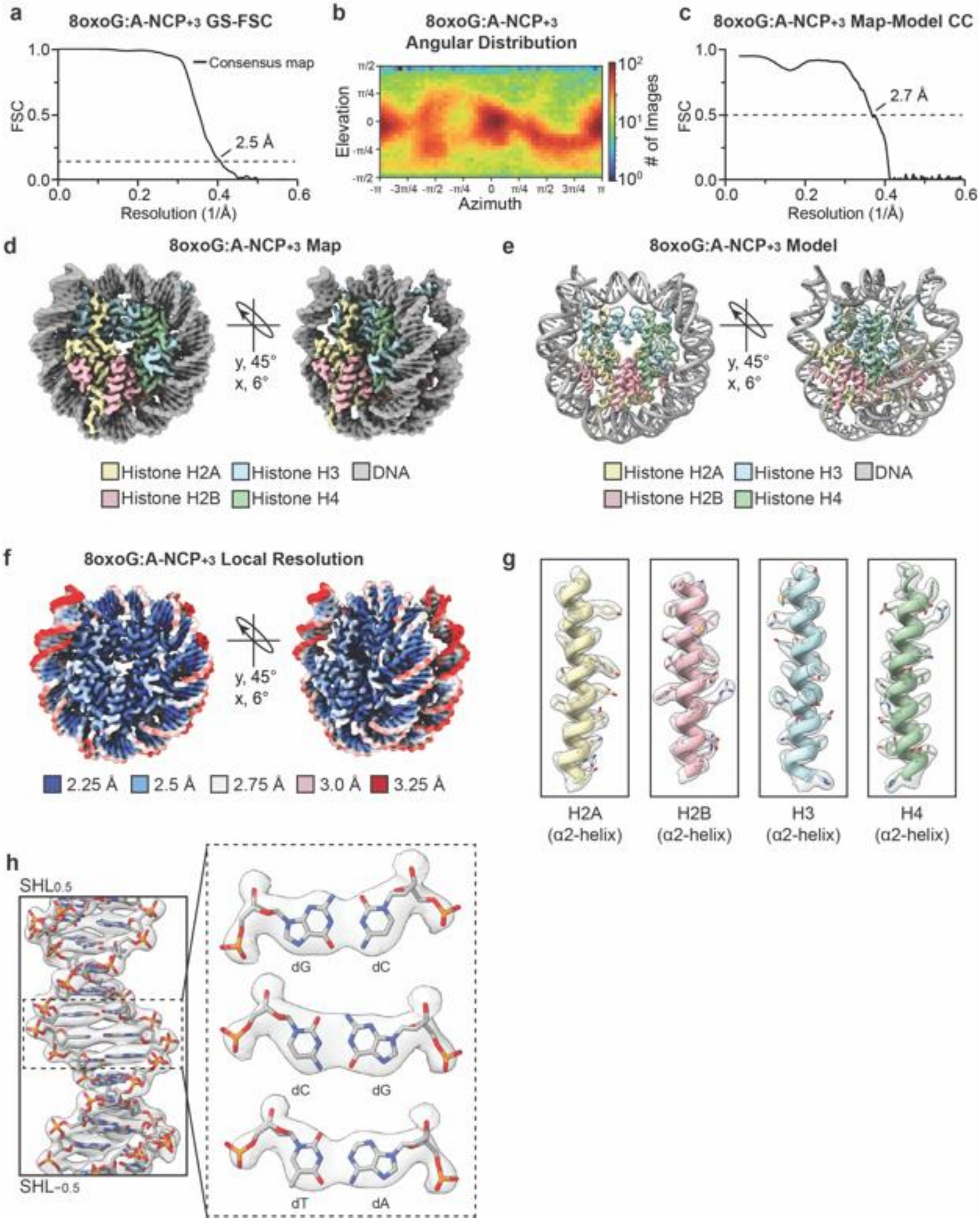

**Supplementary Figure S8: 8oxoG:A-NCP+3 map and model quality assessment**
**a,** Gold-standard Fourier shell correlation (GS-FSC) curve for the 8oxoG:A-NCP+3 cryo-EM map (black solid line). The dashed line corresponds to FSC – 0.143. **b,** Angular distribution heatmap for the 8oxoG:A-NCP+3 cryo-EM map. **c,** Map-to-model FSC curve for the 8oxoG:A-NCP+3 model and 8oxoG:A-NCP+3 cryo-EM map. The dashed line corresponds to FSC – 0.5. **d,** The final 2.5 Å 8oxoG:A-NCP+3 cryo-EM map shown in two orientations. **e,** The final 8oxoG:A-NCP+3 model shown in two orientations. **f,** The local resolution estimation for the 8oxoG:A-NCP+3 cryo-EM map shown in two orientations. **g,** Representative segmented densities for histones H2A, H2B, H3, and H4 in the 8oxoG:A-NCP+3 cryo-EM map. The representative segmented densities from the cryo-EM map are shown as transparent gray surfaces. **h,** DNA base pair densities for base pairs at the dyad in the 8oxoG:A-NCP+2 cryo-EM map. The segmented densities from the cryo-EM map are shown as transparent gray surfaces.

**Supplementary Figure S9**

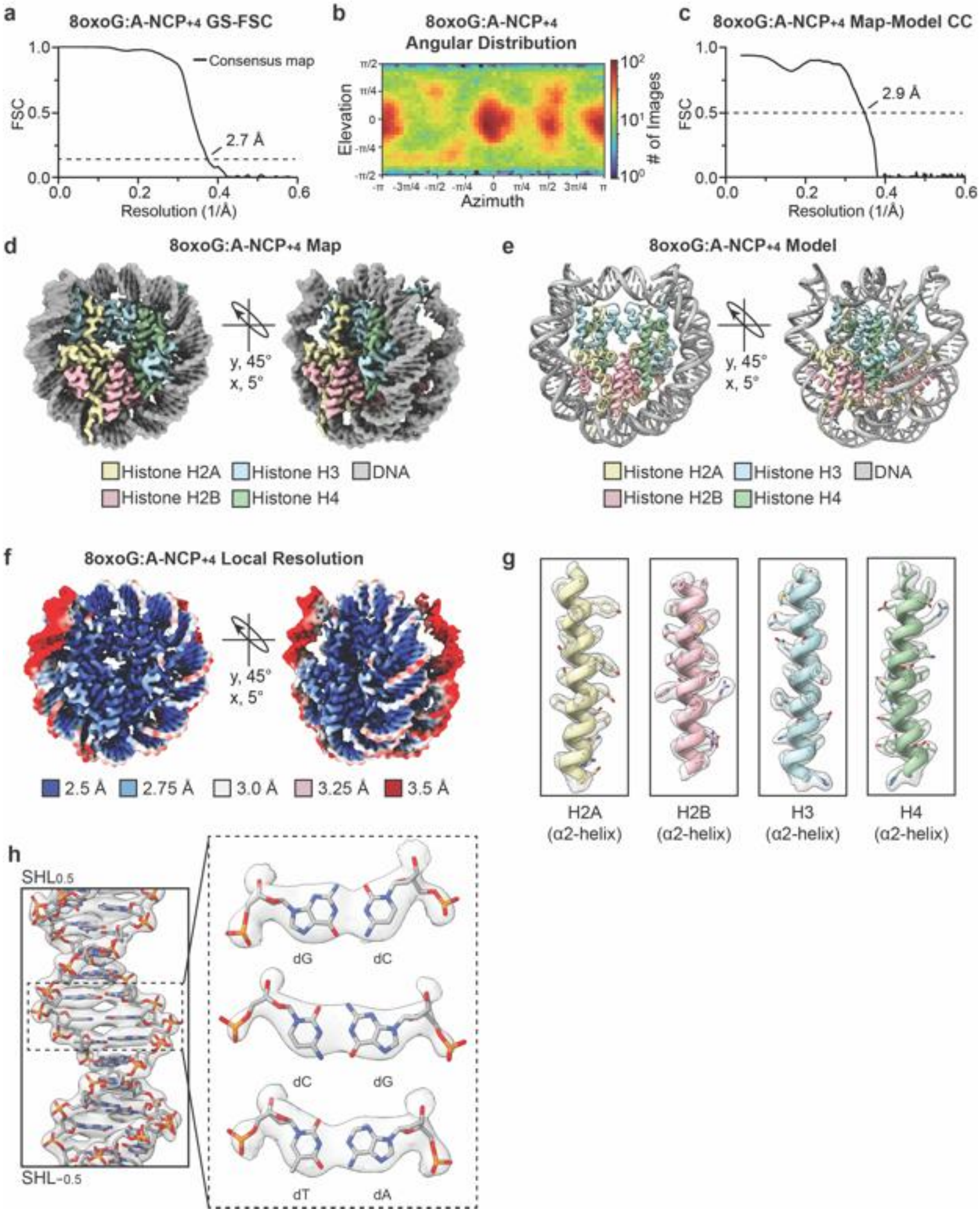

**Supplementary Figure S9: 8oxoG:A-NCP+4 map and model quality assessment**

**a,** Gold-standard Fourier shell correlation (GS-FSC) curve for the 8oxoG:A-NCP+4 cryo-EM map (black solid line). The dashed line corresponds to FSC – 0.143. **b,** Angular distribution heatmap for the 8oxoG:A-NCP+4 cryo-EM map. **c,** Map-to-model FSC curve for the 8oxoG:A-NCP+4 model and 8oxoG:A-NCP+4 cryo-EM map. The dashed line corresponds to FSC – 0.5. **d,** The final 2.7 Å 8oxoG:A-NCP+4 cryo-EM map shown in two orientations. **e,** The final 8oxoG:A-NCP+4 model shown in two orientations. **f,** The local resolution estimation for the 8oxoG:A-NCP+4 cryo-EM map shown in two orientations. **g,** Representative segmented densities for histones H2A, H2B, H3, and H4 in the 8oxoG:A-NCP+4 cryo-EM map. The representative segmented densities from the cryo-EM map are shown as transparent gray surfaces. **h,** DNA base pair densities for base pairs at the dyad in the 8oxoG:A-NCP+4 cryo-EM map. The segmented densities from the cryo-EM map are shown as transparent gray surfaces.

**Supplementary Figure S10**

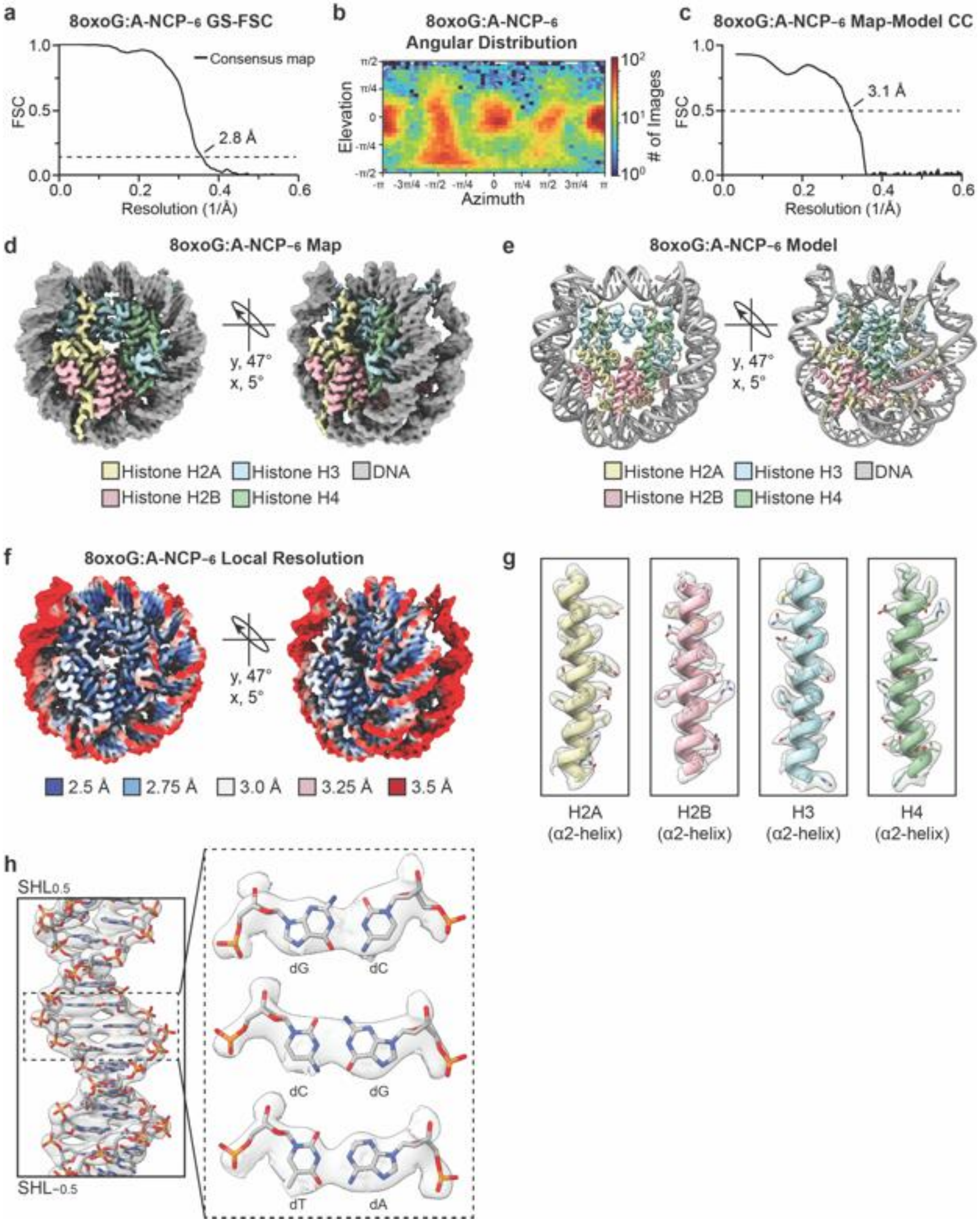

**Supplementary Figure S10: 8oxoG:A-NCP$_{-6}$ map and model quality assessment**

**a,** Gold-standard Fourier shell correlation (GS-FSC) curve for the 8oxoG:A-NCP$_{-6}$ cryo-EM map (black solid line). The dashed line corresponds to FSC – 0.143. **b,** Angular distribution heatmap for the 8oxoG:A-NCP$_{-6}$ cryo-EM map. **c,** Map-to-model FSC curve for the 8oxoG:A-NCP$_{-6}$ model and 8oxoG:A-NCP$_{-6}$ cryo-EM map. The dashed line corresponds to FSC – 0.5. **d,** The final 2.8 Å 8oxoG:A-NCP$_{-6}$ cryo-EM map shown in two orientations. **e,** The final 8oxoG:A-NCP$_{-6}$ model shown in two orientations. **f,** The local resolution estimation for the 8oxoG:A-NCP$_{-6}$ cryo-EM map shown in two orientations. **g,** Representative segmented densities for histones H2A, H2B, H3, and H4 in the 8oxoG:A-NCP$_{-6}$ cryo-EM map. The representative segmented densities from the cryo-EM map are shown as transparent gray surfaces. **h,** DNA base pair densities for base pairs at the dyad in the 8oxoG:A-NCP$_{+4}$ cryo-EM map. The segmented densities from the cryo-EM map are shown as transparent gray surfaces.

**Supplementary Figure S11**

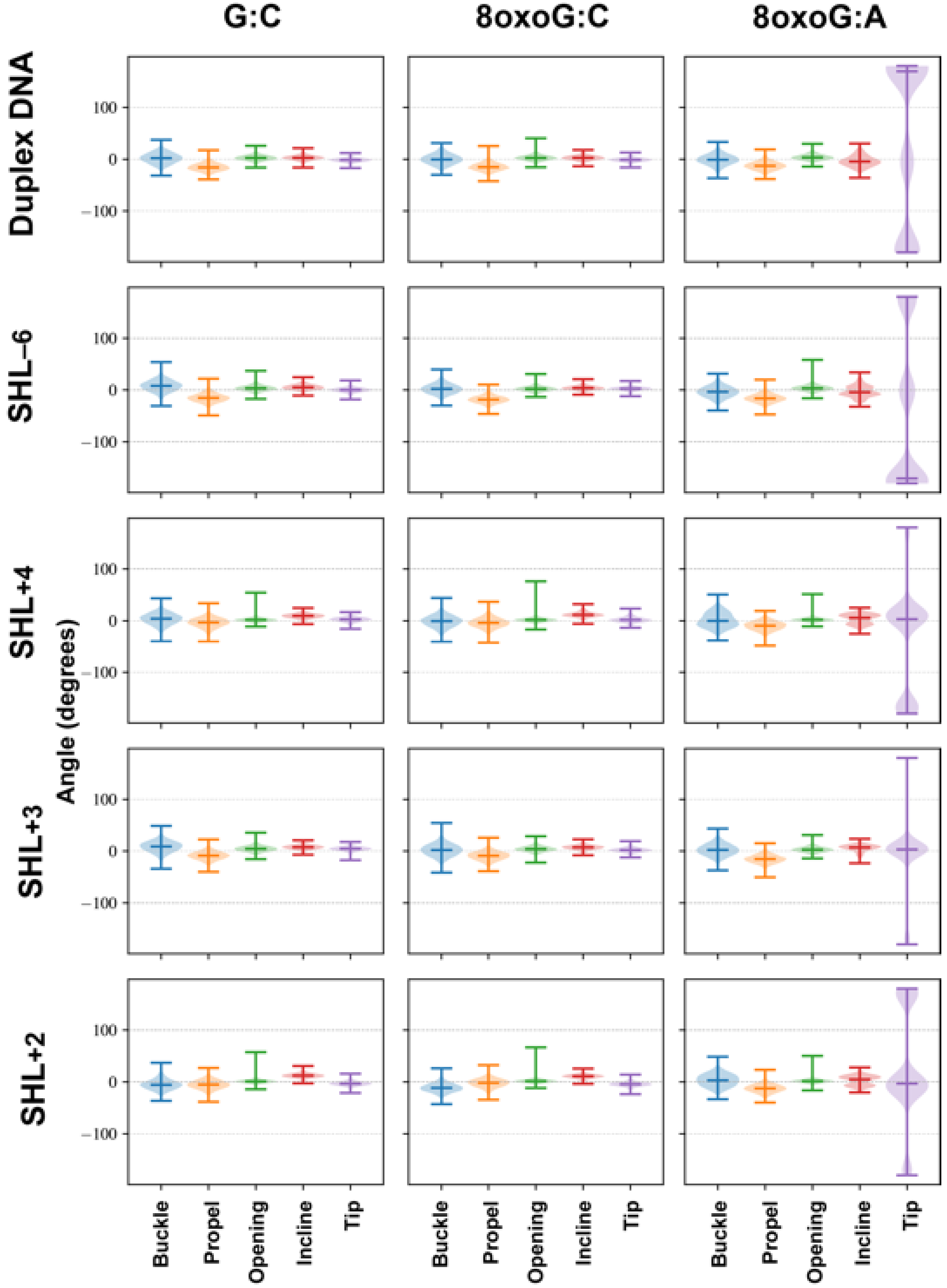


**Supplementary Figure S11: Intra-base pair parameters 5′ of the damage site**
Distribution of intra-base pair angles of the base pair 5′ of the damage site, for the control (left), the 8oxoG:C (center) and the 8oxoG:A (right) systems, in naked DNA or in the nucleosome at the four different SHL.

**Supplementary Figure S12**

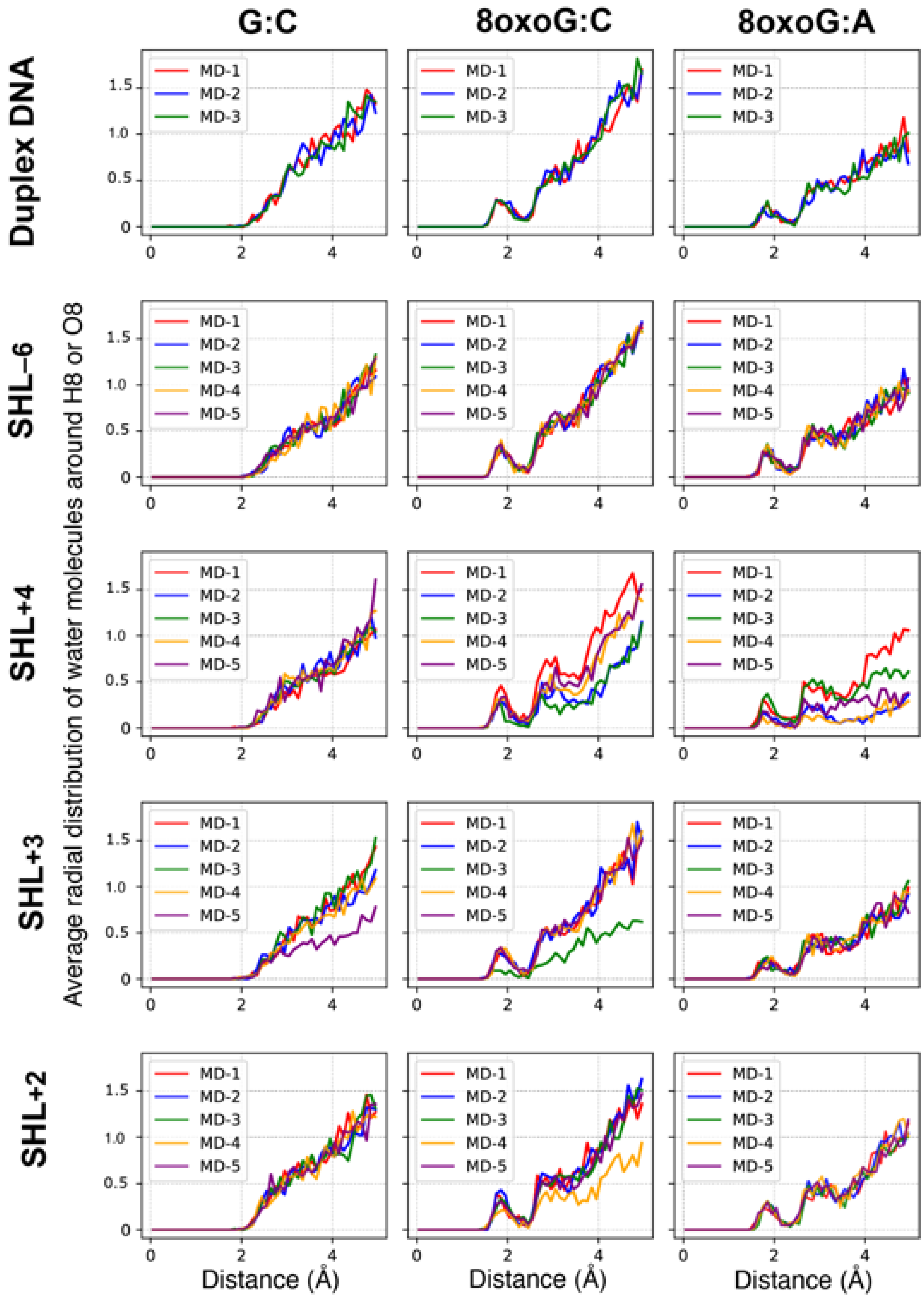


**Supplementary Figure S12: Solvation shell around the 8oxoG O8 atom**
Average radial distribution of the water molecules around the H8 atom in the control (left), or the O8 atom in the 8oxoG:C (center) and 8oxoG:A (right) systems. A first solvation shell is systematically observed around O8.

**Supplementary Figure S13**

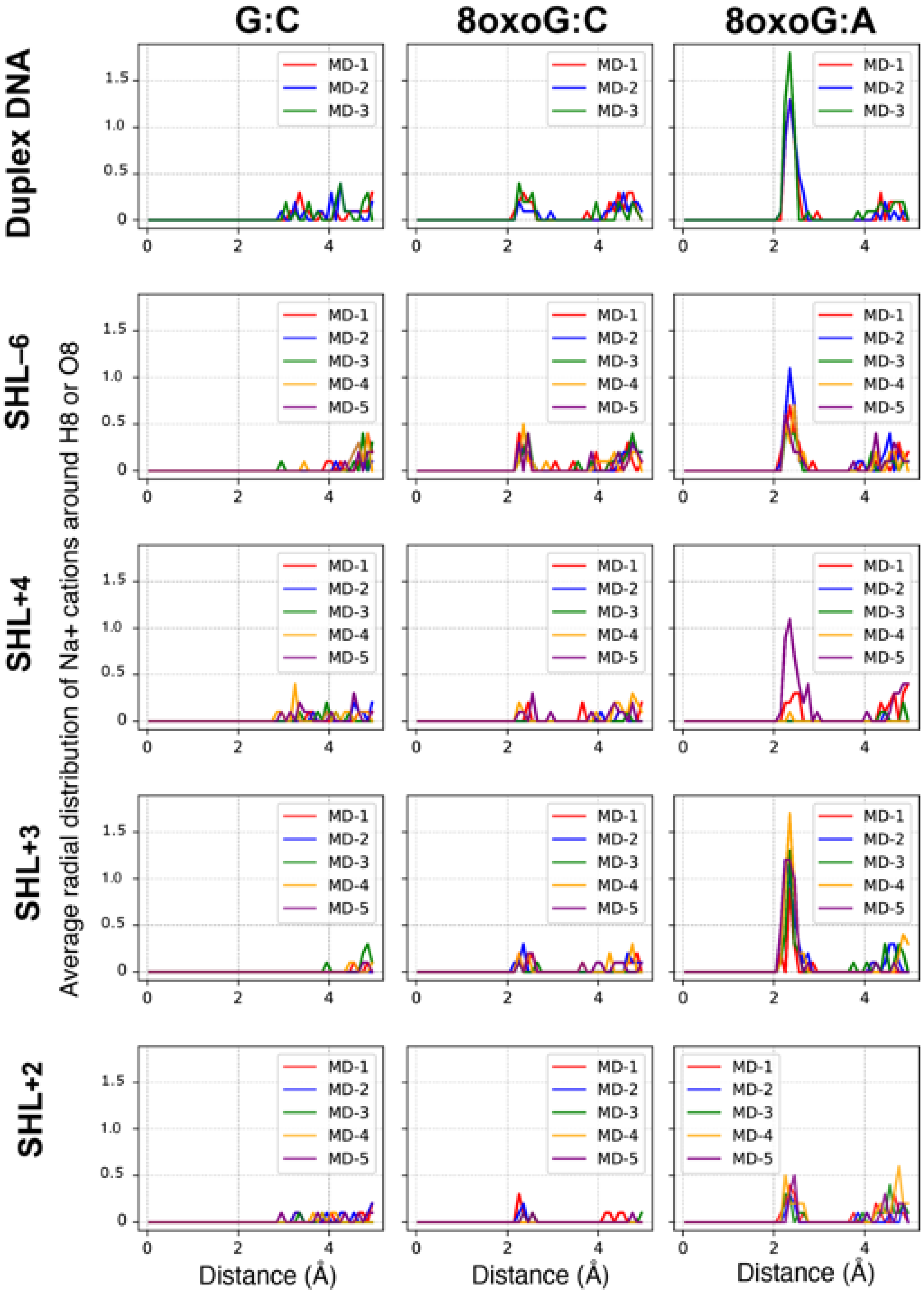


**Supplementary Figure S13: Na$^+$ cations distribution around the 8oxoG O8 atom**
Average radial distribution of the sodium cations around the H8 atom in the control (left), or the O8 atom in the 8oxoG:C (center) and 8oxoG:A (right) systems. The Hoogsteen pairing favors the presence of Na$^+$ around O8.

**Supplementary Figure S14**

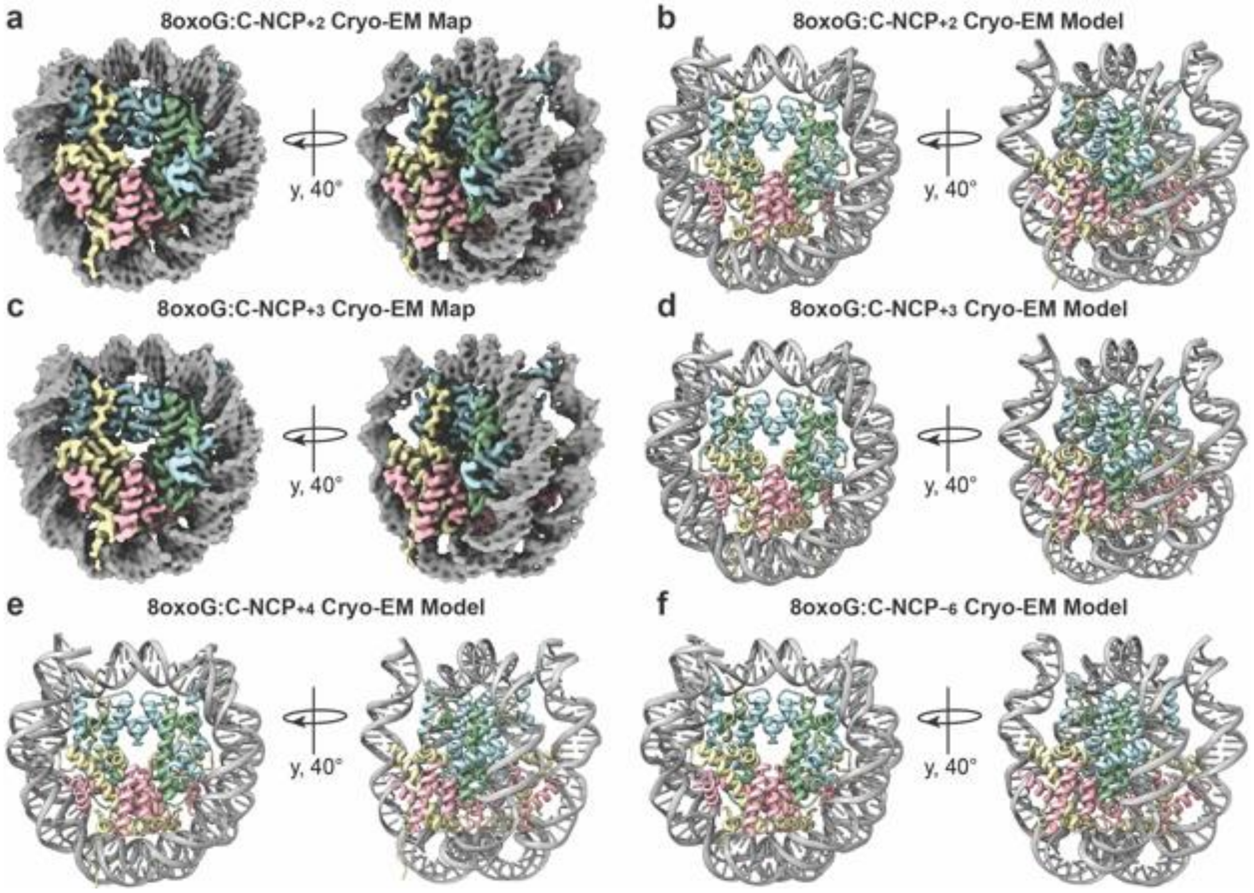


**Supplementary Figure S14: Structural determination of the 8oxoG:C base pair at multiple translational positions in the nucleosome**

Final 8oxoG:C-NCP cryo-EM map and model shown in two orientations at SHL+2 (**a,b**) and SHL+3 (**c,d**). Previously determined models of 8oxoG:C-NCP at SHL+4 (**e**; PDB: 8VWU) and SHL−6 (**f**; PDB: 8VWS).

**Supplementary Figure S15**

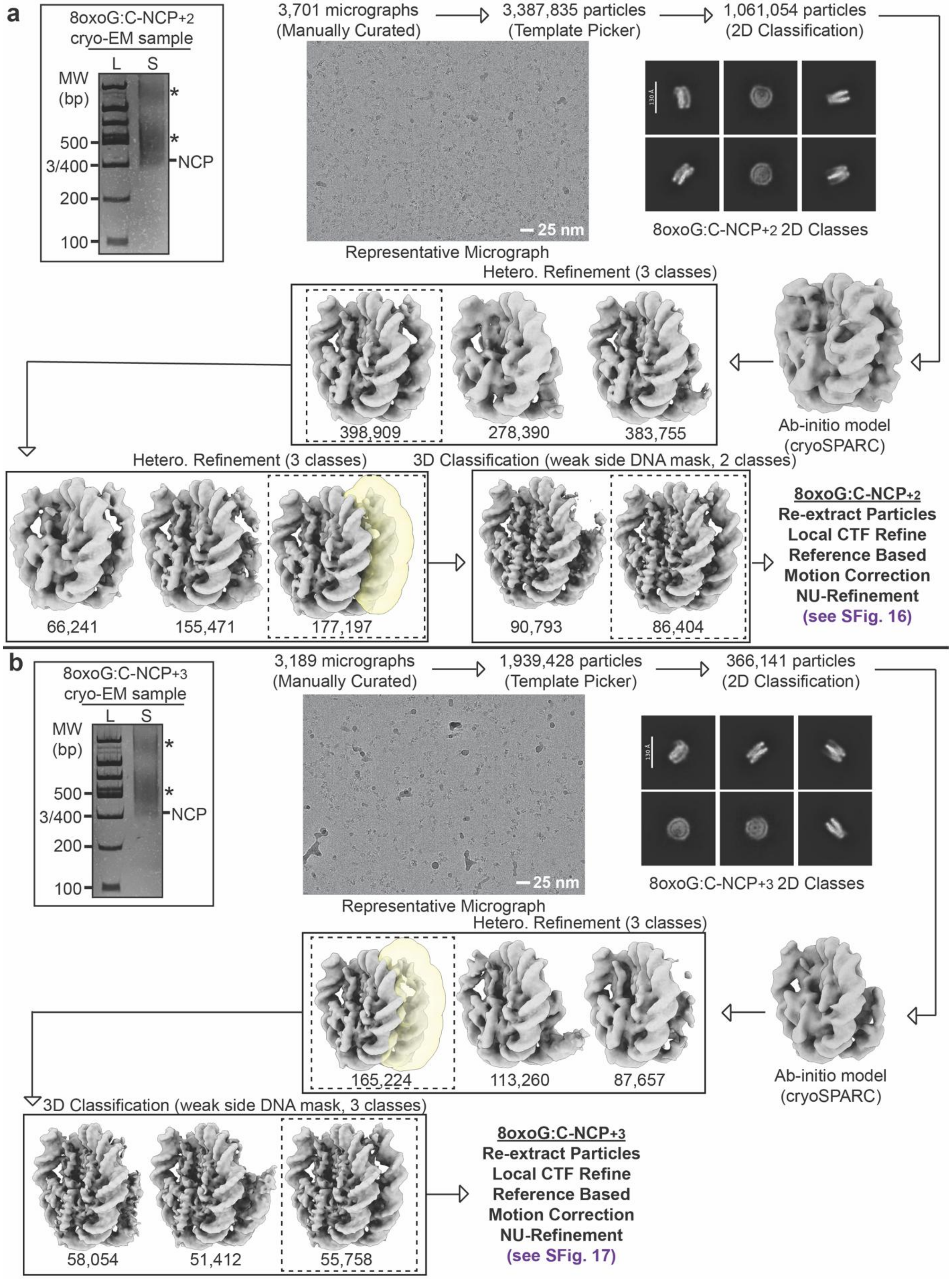

**Supplementary Figure S15: SPA processing workflow for 8oxoG:C-NCP+2 and 8oxoG:C-NCP+3**

**a,** Native PAGE gel of 8oxoG:C-NCP+2 cryo-EM sample and flowchart of the data processing pipeline for the 8oxoG:C-NCP+2 cryo-EM dataset. A 100 bp DNA ladder (L) and the cryo-EM sample (S) are labeled and the NCP was detected using ethidium bromide staining. * indicates higher MW contaminants. A representative micrograph and representative 2D classes from the 8oxoG:C-NCP+2 cryo-EM dataset are shown. The final maps, final models, and quality assessment metrics for 8oxoG:C-NCP+2 can be found in Supplementary Fig. 16. **b,** Native PAGE gel of 8oxoG:C-NCP+3 cryo-EM sample and flowchart of the data processing pipeline for the 8oxoG:C-NCP+3 cryo-EM dataset. A 100 bp DNA ladder (L) and the cryo-EM sample (S) are labeled and the NCP was detected using ethidium bromide staining. * indicates higher MW contaminants. A representative micrograph and representative 2D classes from the 8oxoG:C-NCP+3 cryo-EM dataset are shown. The DNA focus mask used for 3D classification is shown in a transparent yellow surface. The final maps, final models, and quality assessment metrics for 8oxoG:C-NCP+3 can be found in Supplementary Fig. 17.

**Supplementary Figure S16**

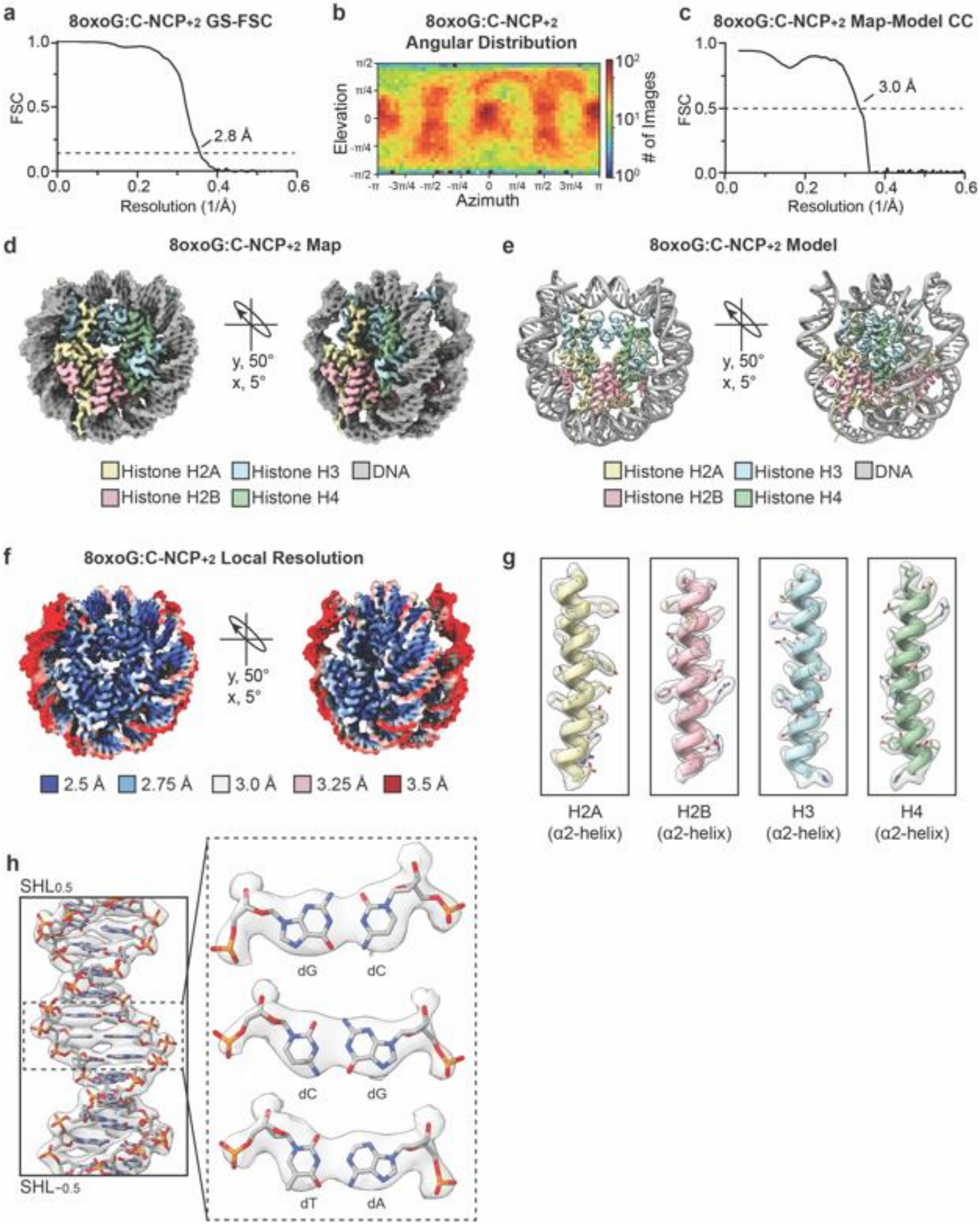

**Supplementary Figure S16: 8oxoG:C-NCP+2 map and model quality assessment**
**a,** Gold-standard Fourier shell correlation (GS-FSC) curve for the 8oxoG:C-NCP+2 cryo-EM map (black solid line). The dashed line corresponds to FSC – 0.143. **b,** Angular distribution heatmap for the 8oxoG:C-NCP+2 cryo-EM map. **c,** Map-to-model FSC curve for the 8oxoG:C-NCP+2 model and 8oxoG:C-NCP+2 cryo-EM map. The dashed line corresponds to FSC – 0.5. **d,** The final 2.8 Å 8oxoG:C-NCP+2 cryo-EM map shown in two orientations. **e,** The final 8oxoG:C-NCP+2 model shown in two orientations. **f,** The local resolution estimation for the 8oxoG:C-NCP+2 cryo-EM map shown in two orientations. **g,** Representative segmented densities for histones H2A, H2B, H3, and H4 in the 8oxoG:C-NCP+2 cryo-EM map. The representative segmented densities from the cryo-EM map are shown as transparent gray surfaces. **h,** DNA base pair densities for base pairs at the dyad in the 8oxoG:C-NCP+2 cryo-EM map. The segmented densities from the cryo-EM map are shown as transparent gray surfaces.

**Supplementary Figure S17**

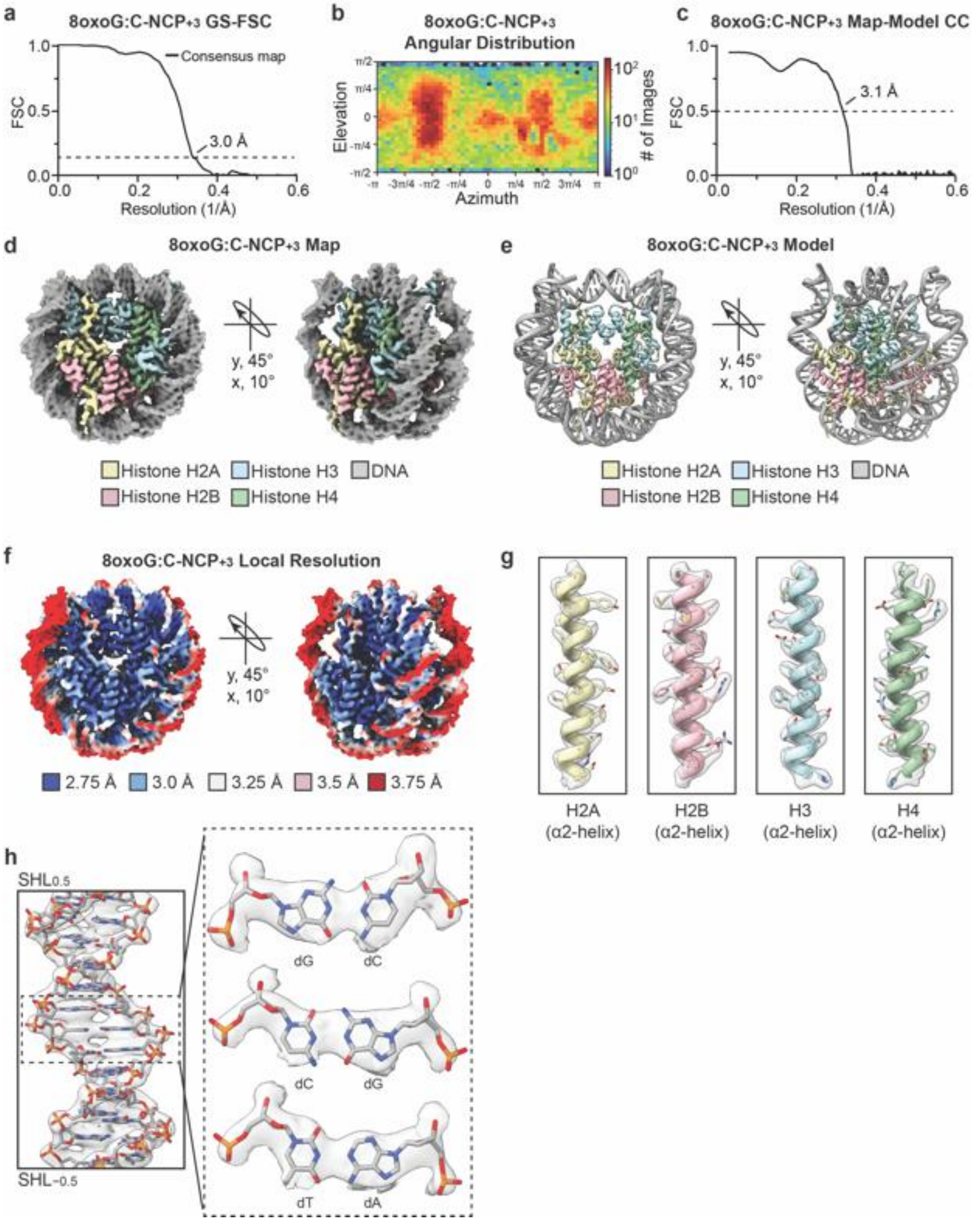

**Supplementary Figure S17: 8oxoG:C-NCP+3 map and model quality assessment**
**a,** Gold-standard Fourier shell correlation (GS-FSC) curve for the 8oxoG:C-NCP+3 cryo-EM map (black solid line). The dashed line corresponds to FSC – 0.143. **b,** Angular distribution heatmap for the 8oxoG:C-NCP+3 cryo-EM map. **c,** Map-to-model FSC curve for the 8oxoG:C-NCP+3 model and 8oxoG:C-NCP+3 cryo-EM map. The dashed line corresponds to FSC – 0.5. **d,** The final 3.0 Å 8oxoG:C-NCP+3 cryo-EM map shown in two orientations. **e,** The final 8oxoG:C-NCP+3 model shown in two orientations. **f,** The local resolution estimation for the 8oxoG:C-NCP+3 cryo-EM map shown in two orientations. **g,** Representative segmented densities for histones H2A, H2B, H3, and H4 in the 8oxoG:C-NCP+3 cryo-EM map. The representative segmented densities from the cryo-EM map are shown as transparent gray surfaces. **h,** DNA base pair densities for base pairs at the dyad in the 8oxoG:C-NCP+2 cryo-EM map. The segmented densities from the cryo-EM map are shown as transparent gray surfaces.

## Supplementary Figure S18

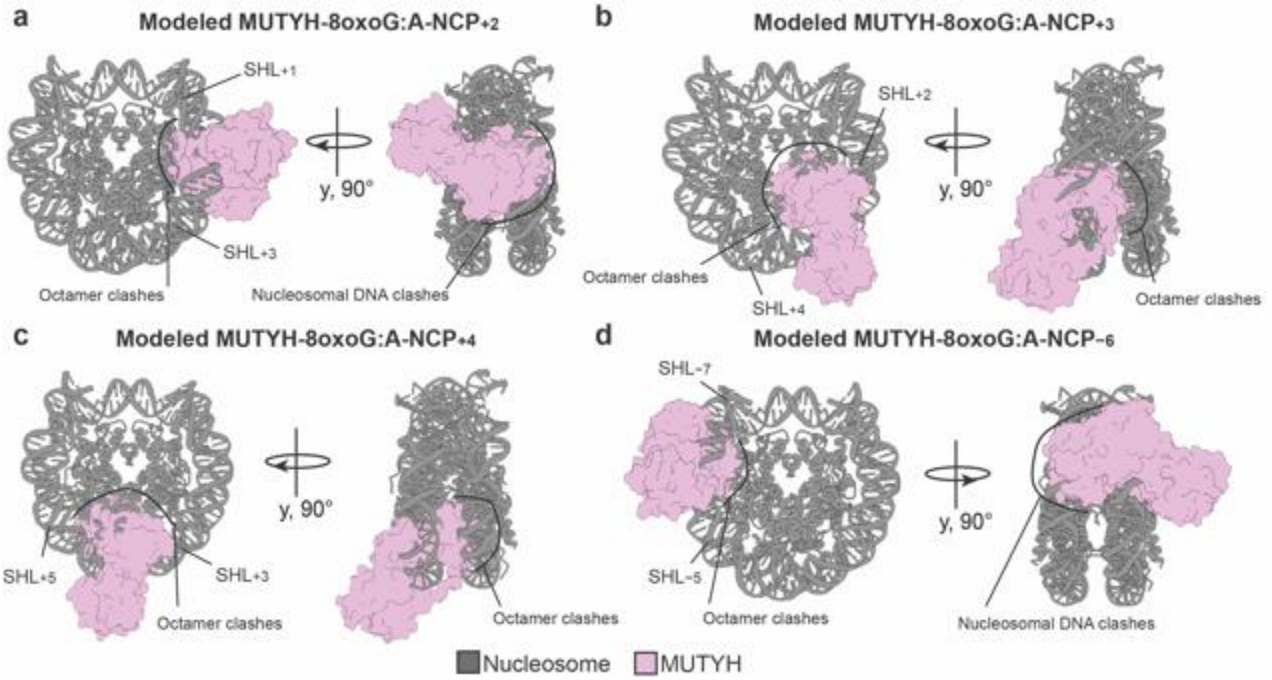


**Supplementary Figure S18: Modeled MUTYH recognition mechanism in the NCP**
Structural models of MUTYH (PDB:6U7T) positioned to interact with 8oxoG:A base pairs at (a) SHL+2, (b) SHL+3, (c) SHL+4, and (d) SHL−6. MUTYH is shown as a surface representation. Significant clashes between MUTYH and the histone octamer and/or nucleosomal DNA are labeled.

## Supplementary Table S1

| SHL of 8oxoG:A | Site of dA on J Strand | Solvent Exposure of dA | Excision of dA from 8oxoG:A by MUTYH | Corresponding cryo-EM Structure |
|---|---|---|---|---|
| –6.8 | 6 | MID | Observed | - |
| –6.4 | 10 | LOW | Observed | - |
| –6.2 | 12 | MID | Not Observed | - |
| –6 | 14 | HIGH | Not Observed | - |
| –5.5 | 19 | LOW | Not Observed | - |
| –5.3 | 21 | LOW | Not Observed | - |
| –4.3 | 31 | LOW | Not Observed | - |
| –4.1 | 33 | MID | Not Observed | - |
| –3.8 | 36 | HIGH | Not Observed | - |
| –3.4 | 40 | LOW | Not Observed | - |
| –3.1 | 43 | MID | Not Observed | - |
| –3 | 44 | HIGH | Not Observed | - |
| –1.5 | 59 | MID | Not Observed | - |
| –1.4 | 60 | LOW | Not Observed | - |
| –1.3 | 61 | LOW | Not Observed | - |
| –1.2 | 62 | MID | Not Observed | - |
| –0.3 | 71 | LOW | Not Observed | - |
| –0.1 | 73 | MID | Not Observed | - |
| 0.6 | 80 | LOW | Not Observed | - |
| 1.6 | 90 | LOW | Not Observed | - |
| 1.7 | 91 | LOW | Not Observed | - |
| 1.9 | 93 | MID | Not Observed | 8oxoG:A-NCP+2 |
| 2.6 | 100 | MID | Not Observed | - |
| 2.8 | 102 | LOW | Not Observed | - |
| 3 | 104 | MID | Not Observed | 8oxoG:A-NCP+3 |
| 3.6 | 110 | MID | Not Observed | - |
| 3.9 | 113 | LOW | Not Observed | - |
| 4 | 114 | MID | Not Observed | 8oxoG:A-NCP+4 |
| 4.2 | 116 | HIGH | Not Observed | - |
| 4.3 | 117 | HIGH | Not Observed | - |
| 4.7 | 121 | LOW | Not Observed | - |

**Supplementary Table S1.** Solvent exposure and MUTYH excision activity of dA sites in NCP 8oxoG:dA mispairs. Solvent exposure was assigned based on structural data.

## Supplementary Table S2

| Data collection and processing | | | | | | |
|---|---|---|---|---|---|---|
| **Dataset** | **8oxoG:C NCP+3** | **8oxoG:C NCP+2** | **8oxoG:A NCP−6** | **8oxoG:A NCP+4** | **8oxoG:A NCP+3** | **8oxoG:A NCP+2** |
| Magnification | 81,000x | 81,000x | 29,000x | 29,000x | 29,000x | 29,000x |
| Voltage (kV) | 300 | 300 | 300 | 300 | 300 | 300 |
| Electron exposure ($e^-/Å^2$) | 60 | 60 | 50 | 50 | 50 | 50 |
| Defocus range (μm) | −0.5 to −2.5 | −0.5 to −2.5 | −0.8 to −2.2 | −0.8 to −2.2 | −0.8 to −2.2 | −0.8 to −2.2 |
| Pixel size (Å) | 0.534 | 0.534 | 0.394 | 0.394 | 0.394 | 0.4125 |
| Symmetry imposed | C1 | C1 | C1 | C1 | C1 | C1 |
| Initial particle images (no.) | 1,939,428 | 3,387,835 | 3,692,093 | 3,732,301 | 4,333,090 | 3,754,627 |
| Final particle images (no.) | 55,539 | 86,030 | 61,519 | 141,630 | 218,657 | 316,231 |
| Map resolution (Å) | 3.0 | 2.8 | 2.8 | 2.7 | 2.5 | 2.5 |
| FSC threshold | 0.143 | 0.143 | 0.143 | 0.143 | 0.143 | 0.143 |
| PDB accession | 10GN | 10GO | 10GJ | 10GK | 10GL | 10GM |
| EMDB accession | EMD-75156 | EMD-75157 | EMD-75152 | EMD-75153 | EMD-75154 | EMD-75155 |
| **Refinement** | | | | | | |
| Initial model used (PDB ID) | 7U51 | 7U51 | 7U51 | 7U51 | 7U51 | 7U51 |
| Model resolution (Å) | 3.1 | 3.0 | 3.1 | 2.9 | 2.7 | 2.7 |
| FSC threshold | 0.5 | 0.5 | 0.5 | 0.5 | 0.5 | 0.5 |
| **Model composition** | | | | | | |
| Nonhydrogen atoms | 12,019 | 12,036 | 11,991 | 12,019 | 12,089 | 12,094 |
| Protein residues | 756 | 759 | 753 | 756 | 764 | 759 |
| Nucleotide | 294 | 294 | 294 | 294 | 294 | 294 |
| **B factors ($Å^2$)** | | | | | | |
| Protein | 86.11 | 104.63 | 113.86 | 95.04 | 108.31 | 108.86 |
| Nucleotide | 157.55 | 166374 | 183.68 | 158.43 | 157.49 | 166.42 |
| **r.m.s. deviations** | | | | | | |
| Bond Length (Å) (# > 4σ) | 0.006 (0) | 0.005 (0) | 0.007 (0) | 0.007 (0) | 0.007 (2) | 0.007 (0) |
| Bond Angles (°) (# > 4σ) | 0.983 (7) | 0.857 (1) | 1.057 (7) | 0.946 (4) | 0.999 (3) | 0.934 (6) |
| **Validation** | | | | | | |
| MolProbity score | 1.35 | 1.36 | 1.67 | 1.39 | 1.41 | 1.43 |
| Clashscore | 3.47 | 4.80 | 5.33 | 3.88 | 4.54 | 4.38 |
| Poor rotamers (%) | 0.00 | 0.00 | 0.00 | 0.00 | 0.00 | 0.00 |
| **Ramachandran plot** | | | | | | |
| Favored (%) | 96.62 | 97.44 | 94.44 | 96.62 | 96.93 | 96.64 |

| | | | | | | |
|---|---|---|---|---|---|---|
| Allowed (%) | 3.38 | 2.56 | 5.56 | 3.38 | 3.07 | 3.36 |
| Disallowed (%) | 0.00 | 0.00 | 0.00 | 0.00 | 0.00 | 0.00 |

## Supplementary Table S3

| Oligo | Sequence (5′– 3′) |
|---|---|
| **8oxoG:C-NCP+3 oligos** | |
| I-strand | ATCGAGAATCCCGGTGCCGAGGCCGCTCAATTGGTCGTAGACA**[8oxoG]**CTCTAG<br>CACCGCTTAAACGCACGTACGCGCTGTCCCCCGCGTTTTAACCGCCAAGGGGAT<br>TACTCCCTAGTCTCCAGGCACGTGTCAGATATATACATCCGAT |
| J-strand | ATCGGATGTATATATCTGACACGTGCCTGGAGACTAGGGAGTAATCCCCTTGGCG<br>GTTAAAACGCGGGGGACAGCGCGTACGTGCGTTTAAGCGGTGCTAGAG**C**TGTC<br>TACGACCAATTGAGCGGCCTCGGCACCGGGATTCTCGAT |
| **8oxoG:C-NCP+2 oligos** | |
| I-strand | ATCGAGAATCCCGGTGCCGAGGCCGCTCAATTGGTCGTAGACAGCTCTAGCACC<br>**[8oxoG]**CTTAAACGCACGTACGCGCTGTCCCCCGCGTTTTAACCGCCAAGGGGAT<br>TACTCCCTAGTCTCCAGGCACGTGTCAGATATATACATCCGAT |
| J-strand | ATCGGATGTATATATCTGACACGTGCCTGGAGACTAGGGAGTAATCCCCTTGGCG<br>GTTAAAACGCGGGGGACAGCGCGTACGTGCGTTTAAG**C**GGTGCTAGAGCTGTC<br>TACGACCAATTGAGCGGCCTCGGCACCGGGATTCTCGAT |
| **8oxoG:A-NCP−6 oligos** | |
| I-strand | ATCGAGAATCCCGGTGCCGAGGCCGCTCAATTGGTCGTAGACAGCTCTAGCACC<br>GCTTAAACGCACGTACGCGCTGTCCCCCGCGTTTTAACCGCCAAGGGGATTACT<br>CCCTAGTCTCCAGGCACGTGTCAGAT**A**TATACATCCGAT |
| J-strand | ATCGGATGTATA**[8oxoG]**ATCTGACACGTGCCTGGAGACTAGGGAGTAATCCCCTT<br>GGCGGTTAAAACGCGGGGGACAGCGCGTACGTGCGTTTAAGCGGTGCTAGAGC<br>TGTCTACGACCAATTGAGCGGCCTCGGCACCGGGATTCTCGAT |
| **8oxoG:A-NCP+4 oligos** | |
| I-strand | ATCGAGAATCCCGGTGCCGAGGCCGCTCAATTG**[8oxoG]**TCGTAGACAGCTCTAG<br>CACCGCTTAAACGCACGTACGCGCTGTCCCCCGCGTTTTAACCGCCAAGGGGAT<br>TACTCCCTAGTCTCCAGGCACGTGTCAGATATATACATCCGAT |
| J-strand | ATCGGATGTATATATCTGACACGTGCCTGGAGACTAGGGAGTAATCCCCTTGGCG<br>GTTAAAACGCGGGGGACAGCGCGTACGTGCGTTTAAGCGGTGCTAGAGCTGTC<br>TACGA**A**CAATTGAGCGGCCTCGGCACCGGGATTCTCGAT |
| **8oxoG:A-NCP+3 oligos** | |
| I-strand | ATCGAGAATCCCGGTGCCGAGGCCGCTCAATTGGTCGTAGACA**[8oxoG]**CTCTAG<br>CACCGCTTAAACGCACGTACGCGCTGTCCCCCGCGTTTTAACCGCCAAGGGGAT<br>TACTCCCTAGTCTCCAGGCACGTGTCAGATATATACATCCGAT |
| J-strand | ATCGGATGTATATATCTGACACGTGCCTGGAGACTAGGGAGTAATCCCCTTGGCG<br>GTTAAAACGCGGGGGACAGCGCGTACGTGCGTTTAAGCGGTGCTAGAG**A**TGTC<br>TACGACCAATTGAGCGGCCTCGGCACCGGGATTCTCGAT |

| **8oxoG:A-NCP+2 oligos** | |
|---|---|
| I-strand | ATCGAGAATCCCGGTGCCGAGGCCGCTCAATTGGTCGTAGACAGCTCTAGCAC C**[8oxoG]**CTTAAACGCACGTACGCGCTGTCCCCCGCGTTTTAACCGCCAAGGGG ATTACTCCCTAGTCTCCAGGCACGTGTCAGATATATACATCCGAT |
| J-strand | ATCGGATGTATATATCTGACACGTGCCTGGAGACTAGGGAGTAATCCCCTTGG CGGTTAAAACGCGGGGGACAGCGCGTACGTGCGTTTAAG**A**GGTGCTAGAGCTG TCTACGACCAATTGAGCGGCCTCGGCACCGGGATTCTCGAT |

**Supplementary Table S3.** DNA oligonucleotides used for reconstitution of NCPs for cryo-EM studies
